# P-selectin glycoprotein ligand-1 (PSGL-1/CD162) is incorporated into clinical HIV-1 isolates and can mediate virus capture and subsequent transfer to permissive cells

**DOI:** 10.1186/s12977-022-00593-5

**Published:** 2022-05-21

**Authors:** Jonathan Burnie, Arvin Tejnarine Persaud, Laxshaginee Thaya, Qingbo Liu, Huiyi Miao, Stephen Grabinsky, Vanessa Norouzi, Paolo Lusso, Vera A. Tang, Christina Guzzo

**Affiliations:** 1 Department of Biological Sciences, University of Toronto Scarborough, 1265 Military Trail, Toronto, ON Canada; 2 Department of Cell and Systems Biology, University of Toronto, 25 Harbord Street, Toronto, ON Canada; 3 Viral Pathogenesis Section, Laboratory of Immunoregulation (LIR), National Institute of Allergy and Infectious Diseases (NIAID), National Institutes of Health (NIH), Bethesda, MD USA; 4 Department of Biochemistry, Microbiology, and Immunology, Faculty of Medicine, Flow Cytometry and Virometry Core Facility, University of Ottawa, Ottawa, ON Canada

**Keywords:** P-selectin glycoprotein ligand-1 (PSGL-1/CD162), P-selectin (CD62P), Human immunodeficiency virus type 1 (HIV-1), HIV-1 infection, HIV-1 envelope, gp120, HIV-1 restriction factors, Virion-incorporated proteins, Virion capture, Calibrated flow virometry

## Abstract

**Background:**

P-selectin glycoprotein ligand-1 (PSGL-1/CD162) has been studied extensively for its role in mediating leukocyte rolling through interactions with its cognate receptor, P-selectin. Recently, PSGL-1 was identified as a novel HIV-1 host restriction factor, particularly when expressed at high levels in the HIV envelope. Importantly, while the potent antiviral activity of PSGL-1 has been clearly demonstrated in various complementary model systems, the breadth of PSGL-1 incorporation across genetically diverse viral isolates and clinical isolates has yet to be described. Additionally, the biological activity of virion-incorporated PSGL-1 has also yet to be shown.

**Results:**

Herein we assessed the levels of PSGL-1 on viruses produced through transfection with various amounts of PSGL-1 plasmid DNA (0–250 ng), compared to levels of PSGL-1 on viruses produced through infection of T cell lines and primary PBMC. We found that very low levels of PSGL-1 plasmid DNA (< 2.5 ng/well) were necessary to generate virus models that could closely mirror the phenotype of viruses produced via infection of T cells and PBMC. Unique to this study, we show that PSGL-1 is incorporated in a broad range of HIV-1 and SIV isolates and that virions with incorporated PSGL-1 are detectable in plasma from viremic HIV-1-infected individuals, corroborating the relevance of PSGL-1 in natural infection. Additionally, we show that PSGL-1 on viruses can bind its cognate selectin receptors, P-, E-, and L-selectins. Finally, we show viruses with endogenous levels of PSGL-1 can be captured by P-selectin and transferred to HIV-permissive bystander cells, highlighting a novel role for PSGL-1 in HIV-1 infection. Notably, viruses which contained high levels of PSGL-1 were noninfectious in our hands, in line with previous findings reporting the potent antiviral activity of PSGL-1.

**Conclusions:**

Our results indicate that levels of PSGL-1 incorporation into virions can vary widely among model systems tested, and that careful tailoring of plasmid levels is required to recapitulate physiological systems when using pseudovirus models. Taken together, our data suggest that PSGL-1 may play diverse roles in the physiology of HIV-1 infection, particularly due to the functionally active state of PSGL-1 on virion surfaces and the breadth of PSGL-1 incorporation among a wide range of viral isolates.

**Supplementary Information:**

The online version contains supplementary material available at 10.1186/s12977-022-00593-5.

## Background

P-selectin glycoprotein ligand-1 (PSGL-1/CD162) is an adhesion molecule expressed on all leukocytes that plays a critical role in the early stages of inflammation due to its ability to bind P-, E- and L-selectins [1–5]. In particular, PSGL-1 has been well-characterized for its involvement in leukocyte recruitement (rolling and tethering) and extravasation into tissues (reviewed in [1, 3, 4, 6, 7]). Stucturally, PSGL-1 is a highly glycosylated homodimeric transmembrane protein, with an extracellular domain (ECD) of 50–60 nm in length that extends far out from the cellular surface [1, 8, 9]. In 2019, Liu et al. were the first to identify PSGL-1 as a novel HIV restriction factor in activated human CD4 + T cells, with inherent antiviral activity through a variety of mechanisms in host cells [10]. Notably, using viruses produced through transfection, Liu et al. showed that when increasing amounts of PSGL-1 plasmid DNA (pDNA) were co-transfected with an HIV-1 infectious molecular clone, virion infectivity was reduced in a dose-dependent manner [10]. Upon further investigation virions were also shown to physically incorporate PSGL-1, and this virion incorporation of PSGL-1 was identified as an additional mechanism to diminish HIV-1 infectivity [10]. Experiments performed by Murakami et al. and Fu et al. in 2020 also demonstrated through knock-down of PSGL-1 in Jurkat cells and CRISPR-mediated KO of PSGL-1 in primary cells, respectively, that physiological levels of PSGL-1 on the cell surface can also result in a modest impairment of particle infectivity [11, 12].

Subsequent studies examining the antiviral effects of virion-incorporated PSGL-1 demonstrated that PGSL-1 could block the binding of virions to target cells [11, 12]. More specifically, it was shown that the large ECD of PSGL-1 was required for this inhibitory effect and that PSGL-1 also has a broad spectrum antiviral effect when ectopically expressed in the envelopes of other viruses, including murine leukemia virus, influenza A and SARS CoV-1 and -2 [11, 13, 14]. Additional characterization of virion-incorporated PSGL-1 revealed a concomitant reduction in the incorporation of the HIV envelope glycoprotein (Env) into virions by sequestering gp41 at the cell membrane [11, 15], providing another measure by which PSGL-1 can restrict virion infectivity. Research on other structurally similar proteins with large extracellular domains has also shown similar inhibitory effects on viral infection and in line with this concept, PSGL-1 was recently reported to be a part of a larger group of antiviral proteins termed Surface-Hinged, Rigidly-Extended Killer (SHREK) proteins [14].

While much has been rapidly uncovered about the antiviral functions of PSGL-1 on HIV-1 and other viruses since its identification as a host restriction factor in 2019 [10], many unanswered questions about the function of PSGL-1 in the physiology of HIV-1 infection remain. Importantly, to tease out the dose-dependent antiviral effects of PSGL-1, many of the experiments characterizing the antiviral functions of this protein have used viruses produced via co-transfection of PSGL-1 and viral constructs in adherent cell lines, although virus from primary CD4 + T cells has also been studied [10–12]. While transfection models are commonly used to produce viruses and express specific host proteins, it is well recognized that the cell type used to produce viruses can alter the composition of cellular proteins incorporated into the virion surface [16–18]. Importantly, when transfecting DNA, the levels of gene expression can be highly variable and can be up to 100-fold higher than natural expression, since gene expression is greatly dependent on the strength of the promoter used [19–21]. It is also important to note that PSGL-1 functionality is strongly dependent on the cell type and activation state of the producer cells, as it requires highly specific glycosylation patterns to bind selectins [3, 4, 7, 22, 23].

While many antiviral properties of PSGL-1 have been characterized to date, it remains unknown whether virion-incorporated PSGL-1 can bind its cognate receptor, P-selectin. This additional binding capacity may impact HIV-1 pathogenesis and provide additional roles for virion-incorporated PSGL-1 in vivo, beyond its current antiviral classification. Indeed, many cellular proteins within the HIV-1 envelope are known to retain their biological function, which can enhance viral infectivity and modify pathogenesis by mediating interactions with other cell surface receptors [16, 24–27]. Furthermore, no studies to date have shown if PSGL-1 is present on circulating strains of viruses in HIV-1 infected patients, which is critical for establishing the clinical relevance of PSGL-1 in HIV-1 infection.

Herein we show that levels of PSGL-1 on virus preparations produced from cells transfected to express PSGL-1 (using 2.5–250 ng of pDNA) are not representative of the level of PSGL-1 present on most primary viral isolates. Additionally, we demonstrate that while PSGL-1 can inhibit the infectivity of viruses generated through transfection in a dose-dependent manner, viruses produced in primary cells remain infectious, despite PSGL-1 incorporation, as detected through antibody-based virus capture assays, immunoblots and flow virometry. Furthermore, to our knowledge, this is the first report to show that PSGL-1 is present on a wide range of HIV-1 isolates produced in primary peripheral blood mononuclear cells (PBMC) and on circulating virions in the plasma of HIV-1 infected patients. Notably, we found that HIV-1 Env (gp120) remains present at detectable levels on all of the primary isolates cultured in vitro, despite PSGL-1’s ability to reduce levels of Env on virions [11, 15]. Most importantly, we demonstrate that viruses displaying PSGL-1 on their surface can be captured with P-selectin (CD62P) and transferred to permissive cells for infection. These data suggest novel roles for PSGL-1 in mediating HIV infection, that further extends the functionality of virion-incorporated PSGL-1 beyond the previously described antiviral activity.

## Results

### Overexpression of PSGL-1 in HEK293T cells markedly reduces infectivity of progeny virions, while viruses produced by natural infection of T cells and PBMC retain infectivity

In our recent work we phenotyped pseudoviruses engineered to display the virion-incorporated cellular proteins integrin α4β7, CD14 and PSGL-1 (CD162). This work led us to the striking observation that PSGL-1 was detected at markedly higher levels on virion surfaces compared to other host proteins, despite all pseudoviruses being produced with equal amounts of pDNA for host protein expression [28]. Since we observed PSGL-1 to be incorporated to a greater extent than other host proteins on the surface of pseudoviruses, we wanted to determine how phenotypically similar viruses produced through transfection were to viruses produced in more physiologically relevant model systems, such as infection of T cell lines and primary cells.

**Fig. 1:**
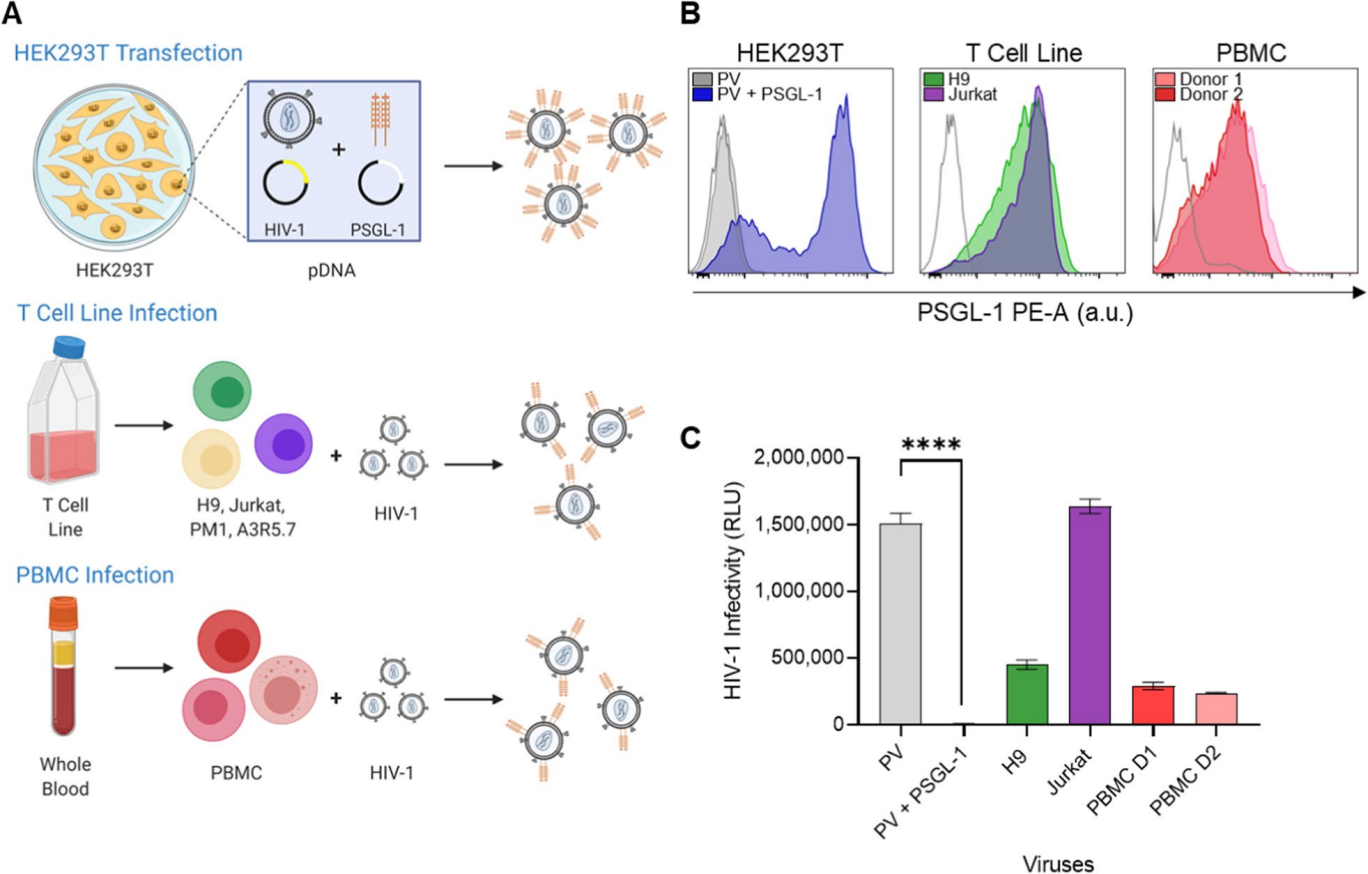
Comparison of PSGL-1 cell surface expression and virus infectivity across model systems. **A** Schematic depicting the three model systems (HEK293T transfection, T cell line infection, PBMC infection) used to produce virus with various amounts of PSGL-1 in the manuscript. **B** Cell surface expression of PSGL-1 on HEK293T cells 48 h after co-transfection with HIV-1 pseudovirus constructs alone (PV; grey histogram), or with pseudovirus constructs and 250 ng of a vector encoding PSGL-1 (PV + PSGL-1, blue histogram). Endogenous levels of PSGL-1 detected by cell surface staining and flow cytometry analysis on the T cell lines, H9 (green) and Jurkat (purple), or activated peripheral blood mononuclear cells (PBMC) from two different donors (red and pink histograms) used for HIV-1 propgation. Isotype staining is shown with unshaded histograms. **C** Viruses produced in the transfected HEK293T cells or HIV IIIB-infected T cell lines and PBMC from **B** were normalized for equal viral p24 input in TZM-bl cell cultures. After 48 h the level of infectivity was measured using luminescence readout (relative light units; RLU). Results displayed are the merged mean ± SEM of three independent experiments with each condition tested in duplicate wells. P values were determined using an unpaired t test (****P < 0.0001)

To this end, we chose to generate viruses with three different cellular models, either through infection or transfection methods (Fig. 1a). We first produced HIV-1 pseudovirus (PV) stocks via co-transfection of HEK293T cells with plasmids expressing an HIV-1 backbone and envelope (SG3^ΔEnv^ backbone + BaL.01 envelope), together with a PSGL-1 expression plasmid (designated as PV + PSGL-1; Fig. 1b). We also generated a matched viral stock without any PSGL-1 (designated as PV; Fig. 1b). For comparison to these viruses produced through transfection, we infected CD4 + T cell lines (H9 and Jurkat) and PBMC from two different donors using IIIB virus stocks and used the progeny viruses for comparative analyses. Before performing downstream analyses of the viruses, we first assessed the levels of PSGL-1 on all of our virus producer cells through cell surface staining with an anti-PSGL-1 antibody and flow cytometry (Fig. 1b). As expected, high levels of PSGL-1 were present on HEK293T cells transfected to express the protein, whereas cells transfected without PSGL-1 pDNA had levels of PSGL-1 that were similar to the isotype staining control. Cells from the H9 and Jurkat T cell lines as well as those from both PBMC donors also displayed appreciable levels of PSGL-1, suggesting that the PSGL-1 was readily available on cells to be incorporated into budding progeny virions. Next we assessed the infectivity of the viruses generated from these different virus production models with the commonly used TZM-bl reporter cell line, using luminescence as a readout for viral infection [12, 29–31]. All virus infections were performed with equal viral inputs (normalized p24 input) to allow for comparisons of infectivity across the virus models. As expected, all of the viruses prepared via infection of T cell lines and PBMC were infectious, with virus produced in Jurkat cells being most infectious. Notably, the pseudovirus produced via transfection without PSGL-1 displayed similar levels of infectivity to the Jurkat virus, while the pseudovirus that was produced in cells which were co-transfected to overexpress PSGL-1 displayed complete abrogation of infection (Fig. 1c).

**Fig. 2:**
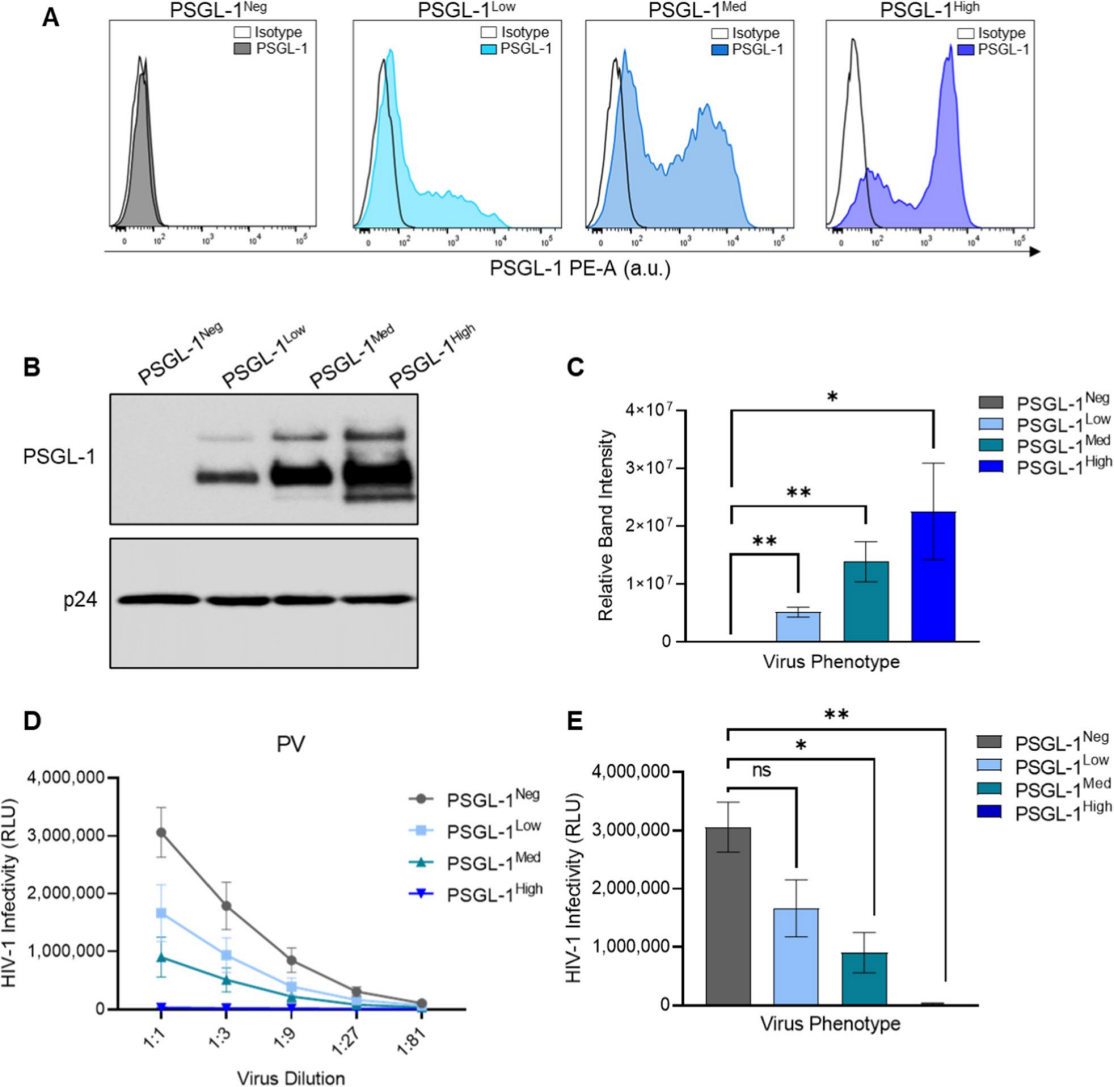
Titration of PSGL-1 expression on virus producing cells and the effect on virus infectivity. **A** Cell surface expression of PSGL-1 on HEK293T cells as detected by flow cytometry 48 h after co-transfection with HIV-1 pseudovirus constructs and increasing amounts of PSGL-1 pDNA (as outlined in Additional file 1: Table S1). Isotype staining is shown with empty histograms, and PSGL-1 staining is shown with filled, coloured histograms (blue or gray). **B** Semi-quantitative comparisons of virion-incorporated PSGL-1 on pseudovirus stocks via immunoblot analysis. The viral capsid protein p24 was used as loading control to ensure equal loading of total virus lysates across all lanes. This immunoblot is representative of three blots performed showing similar results. **C** Densitometric quantitation of immunoblot data from **B**. **D** Virus infection was tested via normalized viral inputs (displayed as 1:1 in graph), followed by three-fold serial dilutions of viruses. All diluted virus stocks were incubated with TZM-bl reporter cells for 48 h before infectivity was measured using luminescence readout (relative light units; RLU). **E** Infectivity from the RLU reading with the most concentrated amount of virus (1:1) is shown. For **C** and **E** the results of unpaired t tests with Bonferroni correction are shown (*P < 0.05; **P < 0.01). Results show the mean ± SEM of three independent experiments

Since many variables could be responsible for the differences in infectivity seen between viruses produced in different producer cells, we designed a more controlled experiment to confirm if differences in PSGL-1 surface expression were responsible for differences in viral infectivity. To this end, we employed a transfection model to simulate a range of different PSGL-1 expression levels on the cell surface of a single virus producer cell type, namely HEK293T cells. As a control, we first produced pseudoviruses completely devoid of PSGL-1 (PSGL-1^Neg^) through co-transfection of only the HIV plasmids, as previously described. For the PSGL-1-containing viruses, in addition to HIV expression plasmids, we also co-transfected different amounts of PSGL-1 pDNA (Additional file 1: Table S1) to yield virus progeny with low, medium and high levels of PSGL-1 in the HIV-1 envelope (designated PSGL-1^Low^, PSGL-1^Med^, and PSGL-1^High^, respectively). Before assessing virus infectivity, we first assesed the cell surface expression of PSGL-1 on the virus producer cells (HEK293T) using flow cytometry (Fig. 2a) As expected the PSGL-1^Neg^ cells did not contain show any PSGL1-1 staining, while the cells transfected to display low, medium and high amounts of PSGL-1 displayed increasing fluorescence intensity as the amount of PSGL-1 pDNA increased. Of particular note, the ‘high’ designation in PSGL-1^High^ virions simply represents the highest amount of pDNA used in our model system, but notably it is an amount of pDNA that is commonly used in our group and others in the field who are studying transient protein expression (250 ng pDNA per well). We also generated viruses with ‘medium’ and ‘low’ levels of PSGL-1 expression, via step-wise reductions in PSGL-1 pDNA (25 and 2.5 ng, respectively).

To verify that our virus production system could generate virions with differential amounts of PSGL-1, we assessed the levels of PSGL-1 associated with total virus lysates using Western blot (Fig. 2b). As expected, increasing amounts of PSGL-1 were observed in the virion lysates that correlated directly with increasing amounts of PSGL-1 pDNA used in the virus preparations (i.e., transfections). This was also apparent through the densitometric quantification of the blots (Fig. 2c).

Next, to compare infectivity of the different virus stocks, TZM-bl cells were incubated with serial dilutions of normalized viral input (Fig. 2d). As expected, the infectivity of all viruses generated through transfection was greatly diminished when PSGL-1 was present at high levels within virions (Fig. 2d; PSGL-1^High^). A dose-dependent potency of PSGL-1 as an antiviral factor was evident in the pseudoviruses, which showed a clear stepwise decline in infectivity as increasing amounts of PSGL-1 pDNA were used to produce virions (Fig. 2d). Remarkably, even the PSGL-1^Low^ pseudovirus, which was created with just 2.5 ng of PSGL-1 pDNA, displayed reduced infectivity compared to PSGL-1^Neg^ pseudovirus (Fig. 2d), suggesting a strong mechanism of selectivity for incorporating this protein into virion envelopes. Statistical analyses of the viral infectivity at the most concentrated virus dilution (1:1) showed significant differences between the control virus without PSGL-1 (PSGL-1^Neg^) and the PSGL-1^Med^ and PSGL-1^High^ viruses (Fig. 2e).

Since immunoblotting only assesses total virion lysates and cannot distinguish proteins that are displayed on the virion surface from the virion interior, our next steps were to employ two other complementary techniques, virion capture and flow virometry, both of which can specifically assay proteins on the virion surface.

### Virions display differential amounts of PSGL-1 and gp120 incorporation dependent on their method of production

**Fig. 3:**
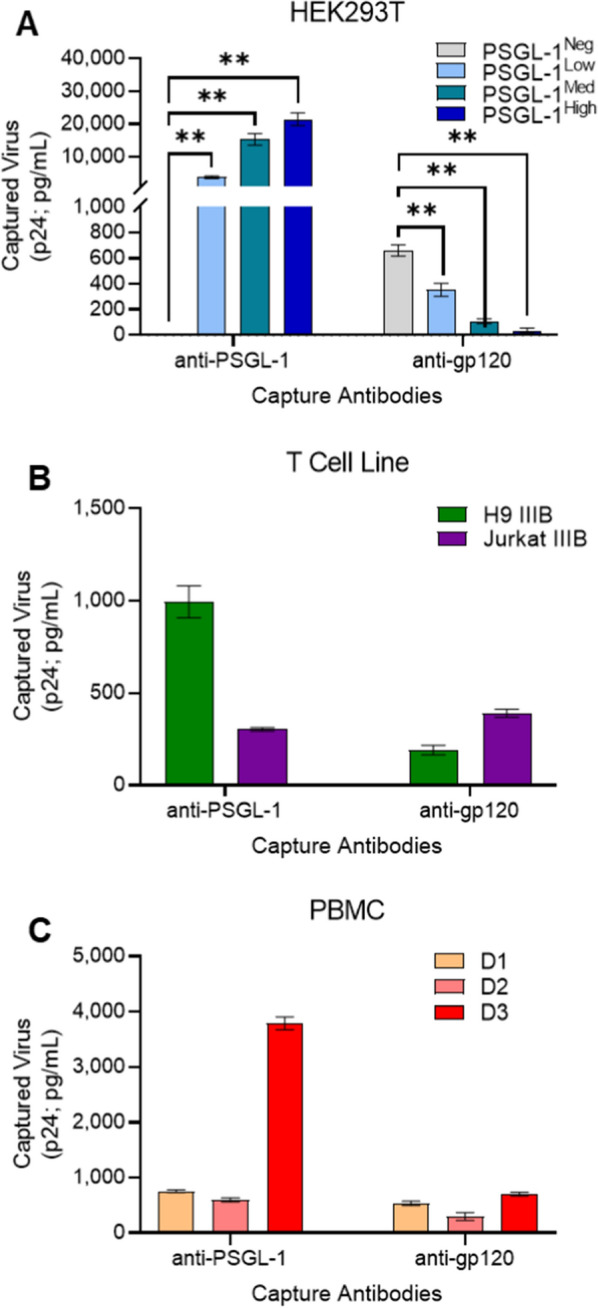
Semi-quantitative comparisons of virion-incorporated PSGL-1 and gp120 on virus stocks via virion capture assay. **A** Virion capture assays were performed with immunomagnetic beads armed with anti-PSGL-1 or anti-gp120 with normalized inputs (5.25 ng of p24 per capture condition) of pseudovirus (HEK293T), **B** T cell line viruses, and **C** PBMC viruses (NL4-3, IIIB, BaL generated in donors 1, 2 and 3 respectively). Bead-associated virus was lysed and HIV-1 p24 Gag was quantified using p24 AlphaLISA as an indicator of the amount of virus capture. Results show the mean ± SEM of three independent experiments in which each condition was tested in duplicate. The results of unpaired t tests with Bonferroni correction are shown (**P < 0.01) for **A**. Levels of background capture as detected using an isotype control antibody were subtracted from the displayed values

To semi-quantitatively assess the amount of PSGL-1 in the envelopes of our different virus preparations, we used a previously described immunomagnetic virion capture assay [24, 28, 32, 33]. This technique was chosen for its ability to selectively assay proteins on the viral surface [14, 34–36]. To compare the relative amounts of PSGL-1 and gp120 present on our range of viruses, we performed antibody-mediated virion capture on viral stocks that were normalized for viral input (equal virus p24) across all viruses tested. We compared virus capture with an anti-PSGL-1 monoclonal antibody (mAb) versus an anti-gp120 mAb (Fig. 3). Through the use of normalized virus inputs across all capture reactions, direct comparisons between the amount of antibody-mediated capture can reflect the relative levels of virion incorporated PSGL-1 and gp120. As expected, the anti-PSGL-1 antibody captured all of the pseudovirus (PV) preparations engineered to contain PSGL-1 (PSGL-1^Low^, PSGL-1^Med^, PSGL-1^High^), while no PSGL-1 capture was present with control viruses (PSGL-1^Neg^) that were devoid of PSGL-1 (Fig. 3a). Stepwise differences in levels of PSGL-1 capture were seen in the PSGL-1-positive pseudoviruses based on their engineered designations (low, medium, high; Fig. 3a). As expected, all of the viruses designed to express PSGL-1 were considered statistically different from the PSGL-1^Neg^ virus. These results were in accordance with what was observed with the immunoblot for PSGL-1 incorporation into pseudoviruses (Fig. 2b). Importantly, virion capture with the anti-gp120 mAb was markedly lower than capture with the anti-PSGL-1 mAb for all pseudovirus phenotypes tested and was particularly low for the PSGL-1^Med^ and PSGL-1^High^ viruses. Indeed, gp120 is normally expected to be present at low levels on circulating virions (8–14 spikes per virion) [37] and PSGL-1 is also known to inhibit Env incorporation into virions [11, 15], so it is unsurprising that gp120 is less abundant on viruses produced from cells transfected to overexpress PSGL-1. In line with this, anti-PSGL-1 capture on viruses produced in T cell lines and PBMC demonstrated that PSGL-1 was present on all of the isolates tested, with a moderate range of variation (Fig. 3b and c). Interestingly, levels of PSGL-1-mediated virion capture were much lower in the more physiologically relevant viruses (from T cell line and PBMC) than pseudoviruses (up to 70-fold less).

While our virion capture assays confirmed that there were differences between the levels of PSGL-1 on viruses produced in different cell types, the technique is only semi-quantitative and reports on the average phenotype of the whole virus sample. Furthermore, since both virion capture and immunoblot are ‘bulk techniques’, they lack the resolution to interrogate a heterogenous sample containing phenotypically distinct virions with individual variation in the amounts of host and viral proteins. To gain more quantitative data on the abundance of PSGL-1 on individual virus particles, we decided to stain virus particles and analyze by flow virometry, for its advantages in providing high throughput, single virion analyses in a calibrated and quantitative readout [38–41].

### Flow virometry analyses indicate that T cell and PBMC viruses incorporate lower amounts of PSGL-1 than viruses produced in cells transfected to express PSGL-1

We have previously provided detailed methodology showing how HIV-1 can be detected by light scatter on sufficiently sensitive cytometers [28, 42], and that virion-incorporated host proteins (including PSGL-1) can be quantified in the HIV-1 envelope of pseudoviruses using flow virometry [28]. More specifically, by using fluorescent and light scatter calibration reference materials along with calibration software, arbitrary light intensities from stained virus samples can be calibrated and expressed in standard units allowing for quantification and comparison of proteins on virions. These standardization methods allow us to draw close estimates of the total number of antibodies bound to each virion, which can be used as a proxy for total number of proteins on individual viral particles [28]. Of note, and in line with the scope of this study, we focused our efforts on PSGL-1 staining and quantitation, and not HIV-1 Env, since the detection of gp120 is currently below our assay threshold with conventional fluorescent labelling.

**Fig. 4:**
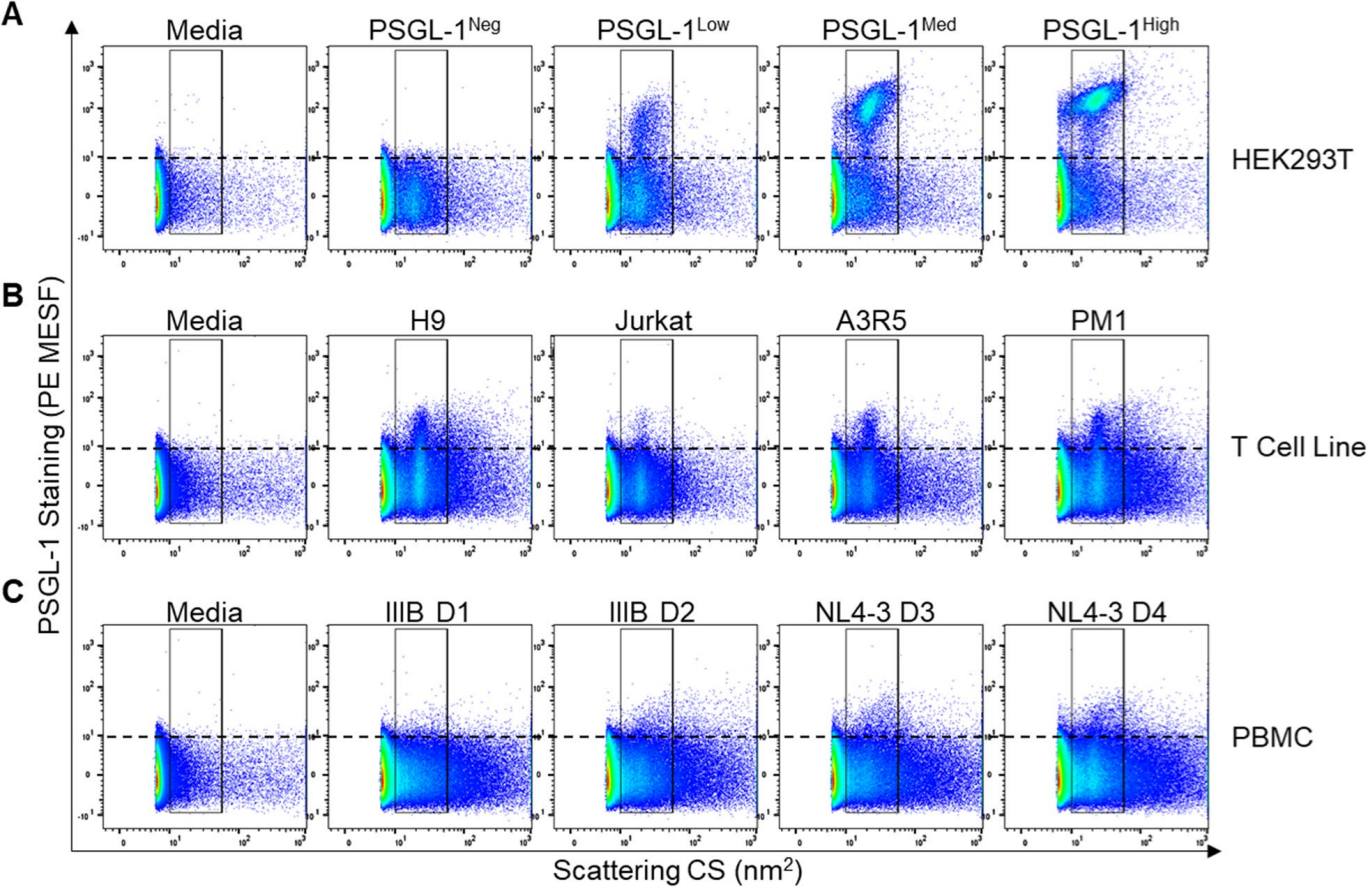
Detecting PSGL-1-incorporation on the surface of viruses using flow virometry. **A** Staining of pseudoviruses produced through transfection of HEK293T cells with different amounts of PSGL-1 DNA (0 ng, 2.5 ng, 25 ng, 250 ng for negative, low, medium, and high phenotypes, respectively) with a PE-conjugated anti-PSGL-1 antibody. The horizontal dotted line on the virus dot plots denotes background fluorescence and the limit of instrument detection (~ 10 MESF). **B** PSGL-1 staining of IIIB viruses produced in the H9, Jurkat, A3R5.7 and PM1 T cells. **C** PSGL-1 staining of HIV IIIB and NL4-3 viruses propropagated in four independent primary cell (PBMC) donors (D1–D4). Data shown are representative of three replicates for each virus stock

To begin, we performed labelling of pseudoviruses that we knew contained high levels of PSGL-1 in the envelope with an R-phycoerythrin (PE)-labelled anti-PSGL-1 mAb (same antibody clone used in virion capture assays). We observed stepwise increasing amounts of median PSGL-1 PE labelling (as reported in calibrated units of molecules of equivalent soluble fluorophore; MESF) on the pseudoviruses generated through transfection (PV; Fig. 4a). The grand median MESF values generated from three biological replicates are shown in Additional file 1: Fig. S1. To determine quantitative MESF staining values, viruses were first gated by side scatter (gate shown in Fig. 4) and then a secondary gate spanning the same SSC profile (not shown) was placed on the population of viruses that were above the threshold of detection (i.e., above background fluorescence; ~ 10 MESF) to generate MESF statistics. This ensured that only viruses that could be visibly distinguished from background signals were contributing to our MESF counts. The pseudoviruses showed a dynamic range of staining, with MESF values as 33 ± 1.2 SD, 93 ± 2.4 SD and 145 ± 2.3 SD (Fig. 4a, top row), for the PSGL-1-Low, -Med and -High viruses, respectively. As expected, control viruses that were devoid of PSGL-1 (PSGL-1^Neg^) did not exhibit positive staining above our limit of fluorescence detection and fell within the instrument background fluorescence. Additionally, a cell culture medium control sample (Media) stained with the same antibody also did not display any appreciable fluorescence.

Having validated that our flow virometry protocol was effective and sensitive enough to detect differences in surface levels of virion-incorporated PSGL-1 in pseudoviruses, we next acquired our T cell line and PBMC viruses (Fig. 4b and c). Since we anticipated that staining endogenous levels of PSGL-1 on viruses produced through infection would be more challenging to detect than staining on our pseudovirus model, we decided to test additional viruses for both model systems (T cell line and PBMC). To this end we included additional IIIB viruses propagated in the A3R5.7 and PM1 T cell lines, since they produced high titre virus that was readily detectable as monodisperse viral populations on our cytometer by light scatter. For viruses produced in primary cells, we increased our sample size to four donors and tested two NL4-3 isolates and two IIIB that were passaged in cells from independent donors. We observed that viruses produced in T cell lines produced more homogenous virus populations than those seen in viruses made in PBMC, as expected given the nature of the culture conditions. T cell line viruses were more monodisperse than viruses propagated in PBMC and produced ‘cleaner’ dot plots with less background attributable to non-virus events. The heterogeneity in PBMC virus scatterplots compared to those of T cell line viruses is likely attributable to differences in extracellular vesicles (EV) produced by cell line versus PBMC cultures (Fig. 4b vs c). Visible PSGL-1 staining was present on all of the viruses produced in T cell lines (Fig. 4b), with the H9 IIIB virus displaying higher levels of staining than the IIIB isolate produced in Jurkat cells, which mirrors the results seen in our virion capture assay (Fig. 3b).

While the majority of the viral isolates produced via infection of T cells and/or PBMC displayed low levels of staining that were visible by eye (Fig. 4b and c), the MESF values were far more modest than those observed on our pseudoviruses, with most reported around the range of 15 MESF (Additional file 1: Figure S1). Notably, the pseudoviruses were produced with stepwise increases in pDNA, with 2.5, 25 and 250 ng of pDNA per well for the Low, Med, and High PSGL-1 viruses. To determine if we could generate viruses through HEK293T transfection that were more similar to the viruses produced through infection, we generated additional virus stocks that were produced through co-transfection with 10- and 100-fold less PSGL-1 pDNA than what was used for the PSGL-1^Low^ virus (0.25 and 0.025 ng; Additional file 1: Figure S2). As expected, these additional viruses displayed much lower levels of PSGL-1 staining, in a range that was more similar to what was seen on viruses from primary PBMC in Fig. 4c.

Since we were able to readily detect PSGL-1 on all three of the PBMC viruses tested using virion capture assays, but lower levels of detection were observed when staining in flow virometry with the same antibody clone (Fig. 4c), we anticipated that PSGL-1 on some primary virions were at levels that were simply too low to detect using our current flow virometry protocols. Indeed, since our capture assays showed that the amount of PSGL-1 on virions produced in T cell lines and PBMC was similar in magnitude to that of gp120 on the viruses tested (i.e., < three-fold difference; Fig. 3c and d), and since we know that anti-gp120 labelling is currently at the cusp of our detection sensitivity for our flow cytometer (roughly ~ 10 PE MESF which is approximately equivalent to ~ 10 molecules per virus) [28], these data are in line with the limit of detection that we would expect using the flow virometry method.

Importantly, our data suggest that contaminating extracellular vesicles that may be present in flow virometry assays do not appear to hinder staining efficiency and/or the reproducibility of our results generated from complementary techniques. However, since vesicles can fall within the range of our virus scatter gate, they may contribute to quantitative assessments ascribed to virus populations. To assess the contribution of extracellular vesicles to our virus populations, we generated matched (mock infected) cell cultures in HEK293T cells, T cell lines and PBMC for comparison to our viruses produced in the same cell types. To this end we transfected HEK293T cells with the same amounts of PSGL-1 pDNA used to generate viruses but substituted viral plasmids for an empty vector. To produce matched controls for viruses produced through infection, T cell lines and primary cells were mock-infected for the same duration of time used in our regular infection protocols (7–10 days). The cell culture supernatants from all three cell types were then stained and acquired as described for the virus preparations (Additional file 1: Figure S3). Unsurprisingly, all of the cell types had extracellular vesicles present from mock infection and/or mock transfection. Cells that were transfected with the highest levels of PSGL-1 pDNA generated the highest levels of PSGL-1-containing EVs. While these vesicles fell within the broad gate that we set for analysis of virus particles, it is important to note that the virus populations displayed different scattering profiles from vesicles, as detected through comparisons of their side scattering profiles with histogram overlays (Additional file 1: Fig. S3). As expected, mock-infected supernatants from T cell lines produced ‘cleaner’ dot plots with lower levels of vesicle staining than what was seen in primary cell cultures, although it should be acknowledged that by simply infecting cells, we expect that the profile of vesicle production would be altered from that of uninfected cells. In summation, while at this time we cannot effectively remove the contributions of EV staining from our virus staining, it is clearly evident that the EV and virus populations are distinct and distinguishable in scatter and staining profiles.

### PSGL-1 is incorporated by a broad range of HIV-1 and SIV isolates and is present on virions in plasma from HIV-infected patients

After demonstrating that there were large differences in the levels of PSGL-1 present on virions produced in different cell types, we next wanted to determine how abundant PSGL-1 was on a broader range of HIV-1 isolates grown in primary PBMC and in clinical samples. Our earlier virus capture experiment had given an indication that levels of incorporation could vary ~ four-fold based on the PBMC donor and viral isolate tested (Fig. 3c), but we sought out to perform a more thorough test. To determine the breadth of PSGL-1 virion-incorporation, we used immunomagnetic virion capture as above to compare PSGL-1 and gp120 incorporation among a panel of lab-adapted and clinical HIV-1 isolates representing different co-receptor usage phenotypes and clades (Table 1), similar to our previous reports for virion-incorporated integrin α4β7 [24]. The different viral isolates were tested undiluted in virion capture assays to determine the potential for PSGL-1 detection at a broad range of viral titres. The PSGL-1 antibody successfully captured all strains of HIV-1 tested, and in most cases at higher levels than anti-gp120 capture. Furthermore, the levels of PSGL-1 capture were independent of HIV-1 clade or co-receptor usage. We compared the relative amount of virion incorporated PSGL-1 to gp120 among the different viral isolates, by calculating the ratio of virion capture with anti-PSGL-1 mAb to anti-gp120 mAb (Table 1, ratio). Across most isolates tested, we observed an appreciable excess of virion-incorporated PSGL-1 relative to gp120, with a ratio average of 4.0 among the 12 replication competent viruses tested. To permit comparisons of these data to the levels of PSGL-1 and gp120 on pseudoviruses, we added the values from our pseudovirus capture to the table (Table 1; HIV-1 PV). In contrast to the PBMC viruses, the ratios on pseudoviruses engineered to be PSGL-1^High^ and PSGL-1^Med^ were 302 and 172, respectively, highlighting several orders of magnitude difference in these model systems for PSGL-1 incorporation. Notably, the PSGL-1^Low^ PV had a ratio that was more similar to that seen with PBMC viruses, demonstrating that physiological levels of PSGL-1 incorporation can be displayed on pseudoviruses produced through transfection when using very low levels of pDNA (< 2.5 ng).

**Table 1:** Virion incorporation of PSGL-1 and gp120 in a panel of clinical and laboratory virus isolates

Virus	Isolate	Clade	Co-receptor usage	Input virus (pg)	Captured Virus		Ratio (PSGL-1:gp120)
					PSGL-1% of input (%)	gp120% of input (%)	
HIV-1	92UG037	A	R5	3113	11	8	1.4
HIV-1	BaL	B	R5	3793	75	37	2.0
HIV-1	ADA	B	R5	8544	81	33	2.5
HIV-1	JRFL	B	R5	565	56	11	5.2
HIV-1	IIIB	B	X4	7068	35	79	0.4
HIV-1	07USLD	B	X4	6492	22	23	1.0
HIV-1	07USLR	B	X4	6035	16	20	0.8
HIV-1	92HT599	B	R5X4	2101	66	26	2.5
HIV-1	93UG065	D	X4	7577	76	56	1.4
HIV-1	93TH057	E	R5	4642	44	8	5.5
HIV-1	CMU06	E	X4	4026	31	4	7.2
HIV-1	97BR019	F	R5X4	9629	31	2	17.5
HIV-1 PV	PSGL-1^Low^	B	R5	35,000	19	1	13.5
HIV-1 PV	PSGL-1^Med^	B	R5	35,000	76	0	172.5
HIV-1 PV	PSGL-1^High^	B	R5	35,000	96	0	302.4
SIV	smE660.307	–	R5	18,035	35	6	5.9
SIV	mac251.745	–	R5	13,044	36	3	11.3

All viruses were produced in activated primary human PBMC, except for the HIV-1 pseudoviruses (PV; produced in HEK293T cells), which are displayed here as controls. The level of virion incorporation was determined by measuring the amount of captured virus by immunomagnetic beads armed with anti-PSGL-1 or anti-gp120 mAbs. Bead-associated virus was lysed and captured virus was quantified by readout of p24Gag for HIV-1 (or p27Gag for SIV). All clinical isolates were minimally passaged in vitro

Notably, we also detected the presence of PSGL-1 at high levels in two SIV isolates (Table 1), providing further support to the notion that PSGL-1 may be a broad-spectrum host restriction factor [11, 13, 14]. Despite the ability for PSGL-1 to sequester gp41 and to disrupt envelope incorporation into virions [11, 15], all of the HIV-1 and SIV isolates tested displayed detectable levels of gp120 capture, above background as determined with capture using an isotype-matched antibody as a negative control. This may suggest that the viral accessory proteins in primary isolates may be sufficient to counteract the inhibitory effects generated by virion-incorporated PSGL-1 and permit higher levels of gp120 incorporation. It is worthwhile mentioning that while the ratio of PGSL-1:gp120 is quite different in our transfection-based virus models versus the infection-based virus models, the total efficiency of PSGL-1 capture was more similar between some of the primary isolates and the PSGL-1^Med^ virus. Similarly, while our pseudoviruses differ greatly from primary isolates in gp120 capture, the level of gp120 capture could vary through the use of a different monoclonal Ab.

**Fig. 5:**
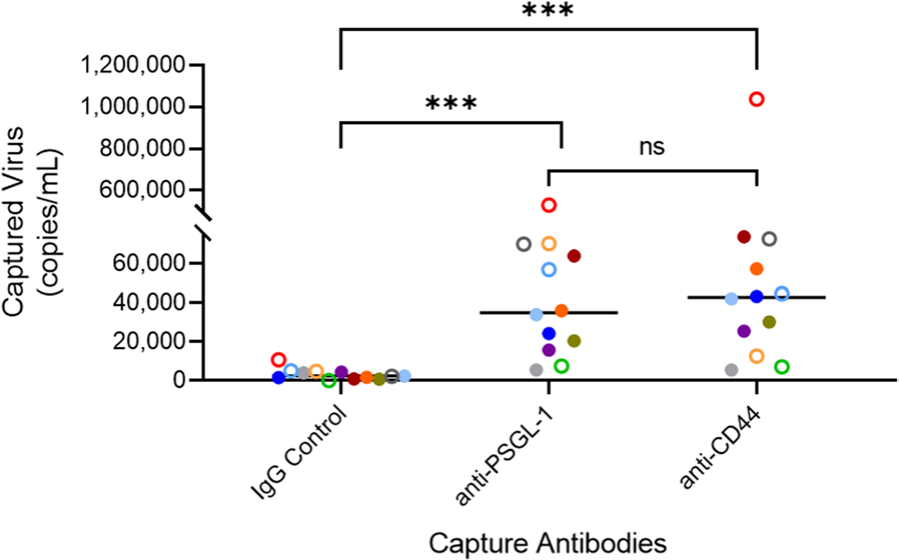
Virions circulating in vivo in HIV-infected patients contain PSGL-1 and CD44. **A** Plasma samples from viremic HIV-infected patients ranging from acute/early to chronic stages of HIV-1 infection were tested in virion capture assays using an isotype control antibody (IgG control), anti-PSGL-1 or anti-CD44. Captured virus was lysed, followed by RNA extraction and quantitative real-time PCR for the detection of HIV-1 genome equivalents (in RNA copies/mL). The sample median for each antibody capture condition is displayed along with results of a Mann–Whitney test (***P < 0.001). Bonferroni correction was used for adjustment of statistical significance. Each unique symbol represents a different patient, with open circles denoting patients in the acute/early stage infection and filled circles as patients in the chronic stage of infection

After determining that virion incorporated PSGL-1 was present on all of the virus stocks produced in vitro, we sought to determine whether PSGL-1 could be identified on virions that circulate in viremic, HIV-infected individuals, as to our knowledge, this has yet to be described in the literature. To this end, we assayed virions in plasma samples from 12 patients at variable stages of HIV infection (acute/early to chronic) using immunomagnetic virus capture (Fig. 5). For this test, we used undiluted virus samples to allow us to observe the range of capture efficiency based on variable viremia levels (Additional file 1: Table S2). To increase the sensitivity and to enhance the success of virion capture from patient plasma samples which contain many inherent factors that can hinder this assay (e.g., plasma proteins, extracellular vesicles, etc.), we employed a slightly modified capture assay using biotin-conjugated antibodies and compatible microbeads, which have shown enhanced levels of virion capture in our hands over the conventional protein-G Dynabead captures used earlier in this study. Among the 12 patient plasmas tested, all patients harbored virus with incorporated PSGL-1 with variable efficiency of incorporation (Fig. 5). As an additional control, we chose to assess CD44 incorporation in parallel in the same plasma samples, as CD44 was previously described to be incorporated with high efficiency into HIV-1 virions [43–45]. CD44 was also incorporated into virions from all patient plasmas tested, and we observed a wide range of virus input recovered by capture with anti-CD44 mAb. Antibodies against CD44 and PSGL-1 were both found to capture virions within patient plasma at levels that were significantly higher than levels of capture with an isotype control antibody. However, no significant differences were seen between the levels of virus capture when targeting either of these two proteins (PSGL-1 or CD44) among the 12 samples tested. We observed a wide range of viral phenotypes and have included the percentage of input virus captured for each respective antibody in Additional file 1: Table S2 for more detailed characterization. Despite the patient variability, PSGL-1 was present on all clinical samples tested, including those from both the acute/early and chronic stages of infection. However, no statistical differences were found between the amount of captured virus between acute and chronic stages of infection within the small size of our study.

### Viruses with incorporated PSGL-1 can be captured by P-selectin and subsequently transferred to target cells for HIV-1 infection

After quantifying virion-incorporated PSGL-1 on a variety of virus types and confirming that PSGL-1 was present on viruses from clinical samples, we next decided to assess whether virion-incorporated PSGL-1 was able to maintain its inherent biological functions and bind its cognate receptors, the selectin family members. We speculated that this was highly plausible, since several other virion-incorporated proteins have been reported to maintain their physiological functions [16, 17, 24, 34, 36, 46, 47]. To begin investigating this, we utilized an infectious molecular clone (IMC) NL4-3 virus produced through co-transfection of the IMC plasmid in HEK293T cells with and without PSGL-1 pDNA (PSGL-1^High^ and PSGL-1^Neg^). We chose to use replication-competent IMC viruses, rather than pseudoviruses, since we anticipated that our downstream viral transfer assay would require multiple rounds of virus replication to detect differences in infectivity caused by P-selectin-mediated capture, which would not be possible with pseudoviruses that cannot replicate. Additonally, for the initial proof of principle test, we chose to test viruses produced with a transfection model, so that we could be certain that the differences we saw in P-selectin binding could be specifically attributed to the definitive presence or absence of PSGL-1. Before using the NL4-3 IMC viruses for binding assays, we validated that there were no major differences between the previously used pseudovirus models and these replication-competent IMC viruses, by performing the same quality control assesment steps as done with all other virus models earlier in this study (i.e., infectivity assays, virion capture, flow virometry; Additional file 1: Fig. S4).

**Fig. 6:**
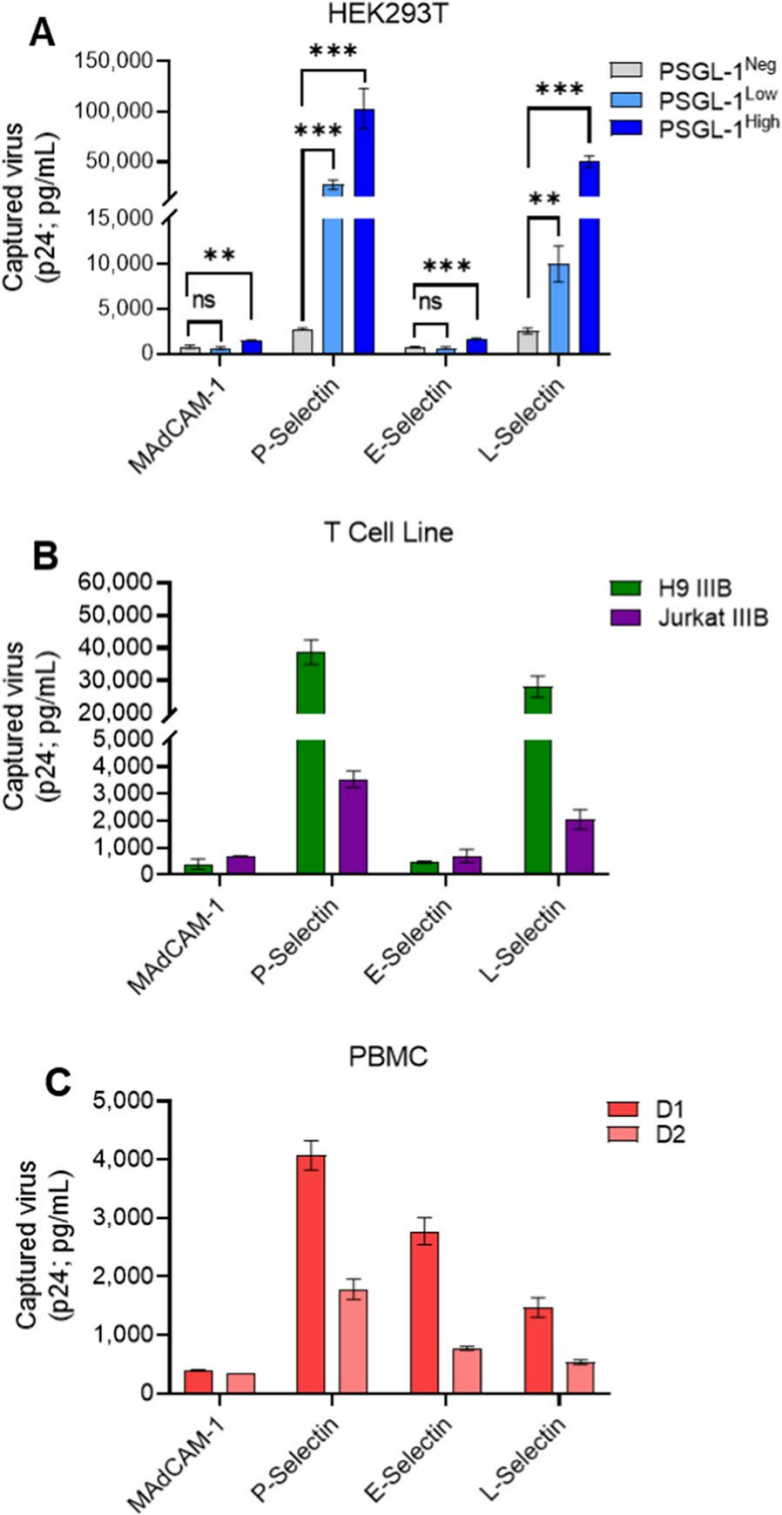
Virion-incorporated PSGL-1 retains the ability to bind selectin family receptors. **A** Virion capture assays were performed with immunomagnetic beads armed with 0.5 μg of recombinant selectins (P-, E-, L-selectin) or MAdCAM-1 with normalized inputs of NL4-3 virus produced through transfection of HEK293T cells with various amounts of PSGL-1 DNA (0 ng, 2.5 ng, 250 ng for negative, low, and high phenotypes respectively). **B** Viruses produced in T cell lines, and **C** PBMC (IIIB generated in two separate PBMC donors, D1 and D2) were added to armed beads at their undiluted titre. Bead-associated virus was lysed and HIV-p24 Gag was quantified using p24 AlphaLISA as an indicator of the amount of virus capture. Results show the mean ± SD of two independent experiments in which each condition was tested in duplicate. The results of unpaired t tests with Bonferroni correction are shown (**P < 0.01, ***P < 0.001)

To determine the ability of viruses to bind to selectin receptors, we coated immunomagnetic beads with recombinant P-, E- and L-selectins and tested whether the armed beads would be able to capture viruses based on interactions with virion-incorporated PSGL-1. As a control we also armed beads with recombinant mucosal vascular addressin cell adhesion molecule 1 (MAdCAM-1), since it is another adhesion molecule that has been shown to interact with the virion-incorporated protein integrin α4β7. From the results we noted that E-selectin yielded low levels of capture for all of the viruses tested produced from transfection, suggesting that PSGL-1 on the virions does not interact with this selectin (Fig. 6a). The levels of E-selectin capture were comparable to what was seen using MAdCAM-1, which does not bind specifically to any proteins on viruses produced in HEK293T cells. However, viruses with low and high levels of PSGL-1 were able to be captured to a much higher extent with both P- and L-selectin (Fig. 6a). Unsurprisingly, P-selectin, the major receptor for PSGL-1, yielded the highest levels of capture in the virus condition with the highest levels of PSGL-1, even with normalized virus input across all capture conditions.

After demonstrating that PSGL-1 on transfection-derived virions could be captured by selectins, we next decided to test capture with T cell line and PBMC viruses, which contained lower levels of PSGL-1. IIIB virus from both the H9 and Jurkat T cell lines displayed similar trends to the PSGL-1 positive viruses produced in HEK293T cells (Fig. 6b). Interestingly, viruses produced in PBMC uniquely showed high levels of E-selectin capture, although this varied greatly between the two PBMC donors tested (Fig. 6c). Due to the highly specific requirements for PSGL-1 binding to its different receptors (e.g., glycosylation, cell activation, etc.) [48–50], it is unsurprising that viruses produced in primary cells showed the widest range of effective binding to selectin receptors.

**Fig. 7:**
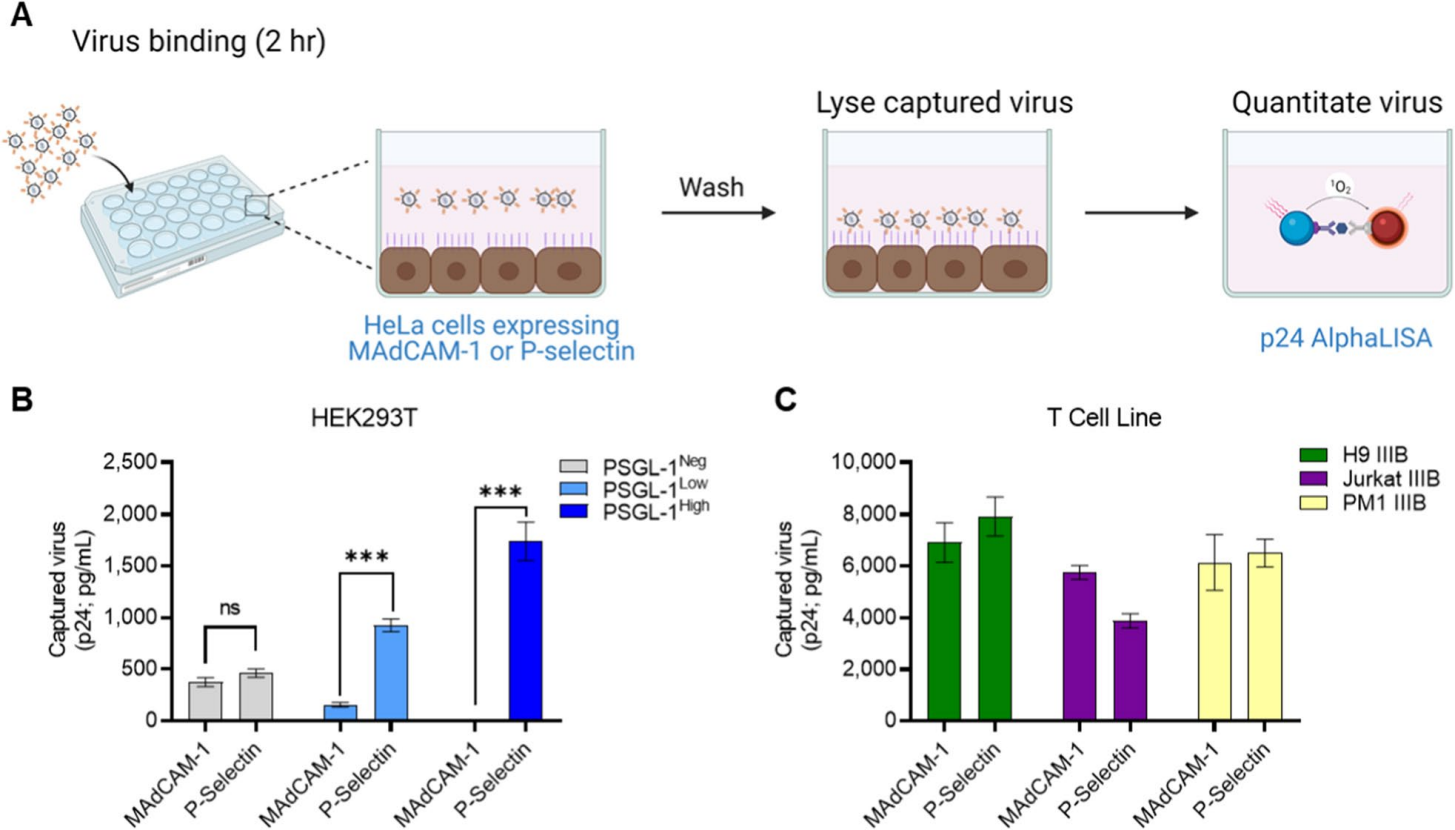
PSGL-1-positive virions can be selectively captured by cells expressing P-selectin. **A** Schematic of cell capture assay: virus preparations were added to wells containing HeLa cells transiently transfected to express MAdCAM-1 or P-selectin to allow for virion capture. Post-incubation, wells were washed extensively to remove unbound virus and were assayed for the amount of captured virus using p24 detection. **B** Experimental results for virus preparations produced through transfection of HEK293T cells with different PSGL-1 phenotypes (0 ng, 2.5 ng, 250 ng of PSGL-1 pDNA for negative, low, and high phenotypes respectively) or through infection (**C**) were used at their undiluted titre. Results displayed are the mean ± SD of two independent experiments with samples tested in duplicate and are representative of three independent experiments. P values were determined using an unpaired t test with Bonferroni correction (***P < 0.001)

Since bead-based virus capture using recombinant proteins has limitations in its physiological resemblance, we decided to test how PSGL-1 capture would occur on a cell expressing cell-surface selectins. For this purpose, we transfected HeLa cells with our capture molecules of interest and assesed the level of captured virus to cell monolayers (Fig. 7a). Since P-selectin is the primary receptor of PSGL-1 and resulted in the highest levels of capture for all of the viruses tested with the recombinant protein experiments (Fig. 6), we chose to compare cell-mediated capture with P-selectin to that of MAdCAM-1 (as a control). As before, we chose to use 293T-derived viruses with no, low or high levels of PSGL-1 (PSGL-1^Neg^, PSGL-1^Low^, PSGL-1^High^) for this test. As expected, the PSGL-1^Neg^ virus which lacks external ectopic adhesion proteins showed no significant differences in the levels of capture with either MAdCAM-1 and P-selectin (Fig. 7a). In contrast to this, both the PSGL-1^Low^ and PSGL-1^High^ viruses showed significantly higher levels of capture with P-selectin compared to MAdCAM-1. While we anticipated a high level of specificity in a virus model that contains few surface proteins with adhesion function, we next decided to test three viruses propogated in T cell lines to see if the same effect would be seen. Notably, all three viruses were able to be captured by the P-selectin and MAdCAM-1 expressing cells with similar efficiency (Fig. 7b), with no clear difference in the specificity of adhesion molecules for capturing viruses produced in T cells. Since there are a wide range of endogenous adhesion factors that are present on viruses produced in T cells that could potentially interact with the HeLa cell surface or with MAdCAM-1 such as integrin α4β7 [16, 24], we were not surprised to find that viruses produced through transfection displayed more stark differences in MAdCAM-1 vs. P-selectin capture.

**Fig. 8:**
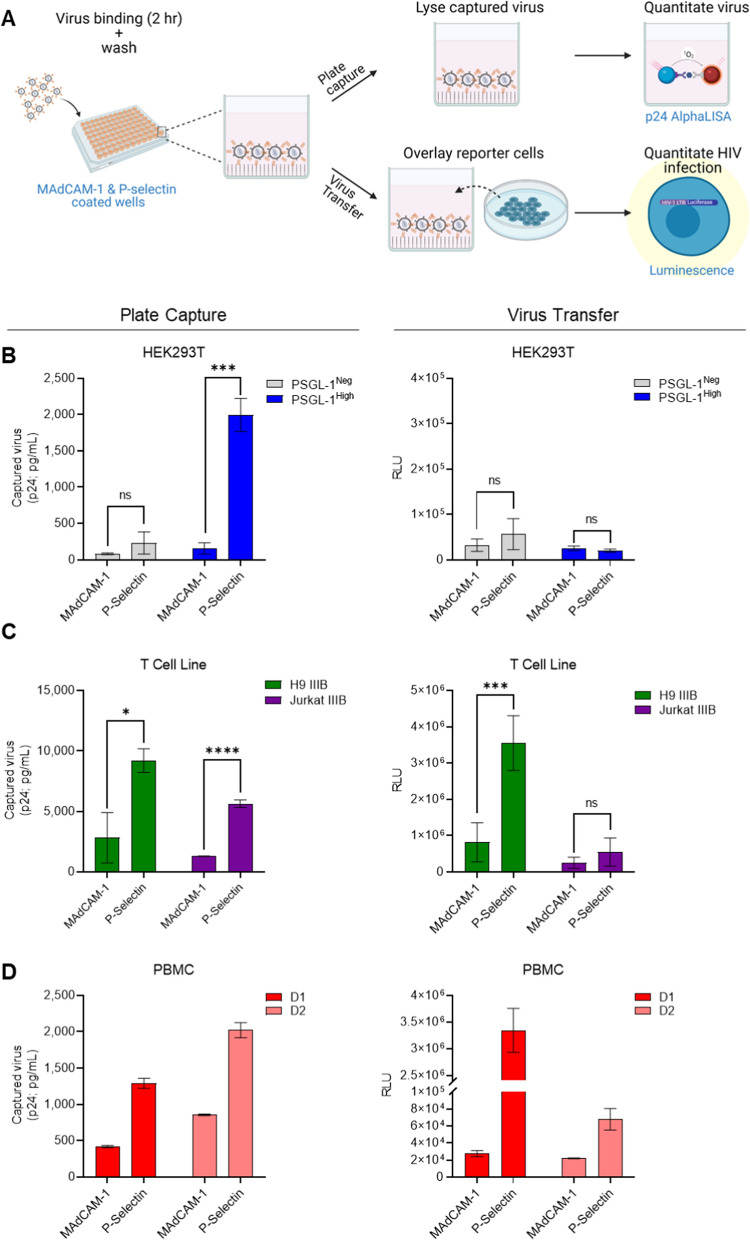
PSGL-1 positive virions can be captured by P-selectin and transferred to HIV-permissive target cells for infection. **A** Schematic depicting the experimental workfolw: virus preparations were added to MadCAM-1 and/or P-selectin coated wells to allow for virion capture. Post-incubation, wells were washed extensively to remove unbound virus and then were either assayed for the amount of captured virus using p24 detection (top workflow), or TZM-bl cells were overlayed onto each well for virus transfer, ad infectivity measurements via luminescence readout (bottom workflow). **B** Experimental results from viruses produced through transfection of HEK293T, **C** infection of T cell lines, and **D** PBMC IIIB viruses in plate-based virus capture (left column) and transfer assays (right column). Results displayed are the mean ± SD of two experiments with samples tested in duplicate wells for **B** and **C**. P values were determined using an unpaired t test (*P < 0.05; ***P < 0.0001). Results shown in **D** are representative of two independent experiments tested with duplicate wells

Our next goal was to determine whether viruses that were captured by P-selectin could be transferred to nearby HIV-permissive cells, since we speculated that this interaction might occur in vivo. To reduce some of the intrinsic specificity problems that can occur when using a cell-based model for capture of viruses, we decided to revert back to a plate-based recombinant capture model to achieve this goal. To this end, we coated wells with either P-selectin or MAdCAM-1 and measured the amount of captured virus in each well by quantification of the viral capsid protein (p24), as illustrated in the top workflow of Fig. 8a. We once again began with our transfection-based virus model to allow for a clear indication of the role of PSGL-1 in this process. We observed high levels of virion capture in the P-selectin coated wells overlayed with PSGL-1^High^ virus, but not in P-selectin coated wells tested with control (PSGL-1^Neg^) virus (Fig. 8b, left panel). Wells with MAdCAM-1 also displayed minimal levels of non-specific virus binding.

Since the primary target of HIV-1 infection, CD4 + T cells, are often found on activated endothelial tissues which display P-selectin in inflammatory conditions [51–53], we were interested in testing whether virions captured by P-selectin could be transferred to nearby permissive cells to elicit infection. We hypothesized that this could suggest new roles for virion-incorporated PSGL-1 in mediating HIV infection, beyond its current scope as an antiviral molecule. As depicted in the workflow schematic in Fig. 8a, we coated wells with P-selectin or MAdCAM-1 to test how these molecules mediate virus capture (top workflow), and then overlaid TZM-bl reporter cells on the wells containing captured virus (after washing away unbound virus) to assess infection 48–96 h later (bottom workflow). As expected, PSGL-1^Neg^ and PSGL-1^High^ IMC viruses showed negligible levels of virus capture-transfer, with a level of background luminescence that is typical of uninfected cells, suggesting that an insignificant level of infection occurred (< 20,000 RLU; Fig. 8b, right panel). These observations were in line with our expectations, since no appreciable levels of PSGL-1^Neg^ virus were captured, and therefore, no virus transfer (or infection) was expected. In the case of the PSGL-1^High^ viruses, while we did observe a high level of P-selectin-mediated capture, we did not expect any virus transfer to permissive cells, since we know these viruses with high levels of PSGL-1 are not infectious, as delineated in previous infectivity assays tested earlier in this study.

Having validated that PSGL-1 on viruses from transfection could be captured by P-selectin and that our model of capture-transfer has a high level of specificity, we next decided to test this model with T cell line and PBMC viruses, which contained lower levels of PSGL-1 and higher levels of gp120. We observed that viruses produced in T cell lines and primary PBMC were captured by P-selectin at levels that were markedly higher than the MAdCAM-1-coated wells (Fig. 8c and d, left panels). Futhermore, captured virus could be transferred to permissive reporter cells for robust detection of infection (Fig. 8c and d, right panels). We also observed that virion capture and transfer with virus produced in Jurkat cells occurred at lower levels than those seen with the same viral isolate produced in H9 cells; however, this finding displayed a similar trend as observed earlier with anti-PSGL-1 antibody capture assays and flow virometry on these viruses (Figs. 3 and 4). Most importantly, both PBMC viruses were effectively captured by P-selectin and transferred to HIV-permissive cells, suggesting that this mechanism of PSGL-1-mediated virus capture and transfer might also occur when HIV viruses encounter P-selectin on cell surfaces in vivo.

As an additional test to confirm that the plate-based virion capture seen using recombinant P-selectin was not simply caused by non-specific binding of virus to the adhesion protein, we tested the T cell line viruses in a similar capture-transfer assay whereby we substituted the capture molecule of P-selectin with antibodies specific for PSGL-1, gp120 or a non-specific isotype control (Additional file 1: Fig. S5). We were impressed to find highly reproducible sensitivity of the capture-transfer mechanisms, whereby virions were captured with the anti-PSGL-1 antibody and transferred to permissive cells with similar efficiency as that seen with recombinant P-selectin capture. Also, the relative levels of plate-based capture between anti-PSGL-1 and anti-gp120 were similar to those previously observed in virion capture assays earlier in this study (Fig. 3). Taken together, these data provide three validations of our capture-transfer model system: (1) that the capture-transfer mediated by P-selectin is reproducible even when using different capture molecules targetting the same protein (anti-PSGL-1 vs. recombinant-P-selectin); (2) that our assay model can be reliably applied to capture with other virion-incorporated proteins, like gp120; and (3) that our capture-transfer model system can reflect differences in the level of virion-incorporated proteins, as we observed less capture-transfer when capturing with anti-gp120, which is less abundant on virions.

## Discussion

While PSGL-1 was first identified as a host restriction factor in a proteomic screen of activated primary T cells [10], many of the subsequent experiments characterizing the antiviral properties of the protein have been performed with cells transfected to ectopically express PSGL-1 [11–15]. While transfection models are valuable tools in characterizing the antiviral properties of restriction factors, we show that these kind of PSGL-1 expression models can overestimate the restriction effect of PSGL-1, depending on the level of pDNA used. Furthermore, these models are not always representative of biologically-relevant viruses produced by natural infection of T cells and primary PBMC. While endogenous levels of PSGL-1 on virions can also reduce infectivity [11, 12], when using transfection systems to study the impacts of PSGL-1 (and other host molecules) on viruses, it is important for researchers to tailor transfection systems to resemble the wide range of variation that can occur under physiological conditions of virus production. An example of that is demonstrated herein using our PSGL-1^Low^ virus, which was the most similar virus model to the more biologically relevant viruses (T cell line and PBMC viruses).

Our work shows that virions produced from T cells and PBMC remain highly infectious, with more virion-incorporated gp120, and markedly lower amounts of virion-incorporated PSGL-1, particularly when compared to our PSGL-1^High^ virus which was generated through transfection to overexpress PSGL-1. Unique to this study, our observations were made with both semi-quantitative techniques, which assessed averages of virus populations, and flow virometry, which can phenotype individual virions with high sensitivity [41]. Using orthogonal techniques, we report consistent results showing low-moderate levels of PSGL-1 on primary viruses, with comparably high PSGL-1 levels on viruses produced through transfection, corroborating the notion that different virus production systems can lead to very distinct virus phenotypes. In line with this, in order to create a virus model via transfection that matched the levels of PSGL-1 detected on viruses from PBMC donors, we needed to reduce the level of pDNA typically used for ectopic host protein expression by over 250-fold from standard experiments in our previous studies. Indeed, initial work characterizing the antiviral properties of PSGL-1 utilized a wide range of pDNA (0–600 ng) to investigate the dose–response of infectivity in the presence of PSGL-1 [11, 15], which informed our selection of the range of pDNA values used in this study (0–250 ng). Of note, using calibrated flow virometry (PE MESF) we were able to estimate that the number of PSGL-1 molecules on the pseudovirus preparations were as low as ~ 35 and as high as ~ 145 proteins per virion on the PSGL-1^Low^ and PSGL-1^High^ viruses, respectively. However, even our PSGL-1^Low^ pseudovirus still yielded quantitative staining values that were double what was present for viruses grown in primary cells, highlighting the inherent difference in these model systems. Despite this, the values for the PSGL-1^High^ pseudoviruses are in a similar range to those which our group has previously reported on PSGL-1 + pseudoviruses [28], indicating that flow virometry is a reproducible technique for protein quantification on virions. While we acknowledge the current limitations of flow virometry techniques and caution that MESF values are estimations and not absolute quantifications, it is very likely that the number of PSGL-1 proteins on viruses produced in PBMC is less than 10–20 molecules per virion, since all of our viruses fell within this range. Optimizing the threshold for detection of low abundance antigens, namely PSGL-1 and gp120, on virions produced in primary cells and patient samples using flow virometry remains the scope of future work to be performed by our group. While the importance of extracellular vesicles in HIV infection continues to be uncovered, the presence of these particles in flow virometry remains a challenge in the field [54–56] that requires future attention and protocol development. However, our flow virometry results reported herein were consistent with results from complementary techniques, including our virion-capture assay. Since our capture assay employs a final readout for the viral capsid protein (p24), it minimizes the bias that contaminating EVs may add, as others have shown very low levels of p24 associated with EVs [56, 57].

It is important to mention that while the addition of PSGL-1 to the HIV envelope has been shown to decrease levels of gp120 within virions in vitro [11, 15], all of the viruses produced in PBMC were shown to have detectable levels of gp120, despite the presence of PSGL-1. Interestingly, this was not the case for viruses produced in transfection, further reinforcing the differential outcomes that different model systems can produce.

In this study we found that viruses propagated in T cell lines were much more similar to viruses passaged in primary PBMC, rather than those produced through transfection of HEK293T cells. Although the antiviral role of PSGL-1 is known to be antagonized by the viral accessory proteins Vpu and Nef [10–12, 15] in addition to HIV Gag, it is possible that additional counteracting factors may be at play in viruses produced via infection of primary cells (and T cell lines). Though clear differences were apparent in the PSGL-1:gp120 ratios of pseudoviruses and PBMC viruses produced in vitro, the fact that PSGL-1 was incorporated in all of the PBMC-produced virus isolates tested, including SIV strains, demonstrates the biological importance of PSGL-1 in HIV infection and also supports the idea that PSGL-1 may play a role in the pathogenicity of a many enveloped viruses [11, 13, 14].

While the glycosylation of PSGL-1 is known to be cell-type dependent and critical for the ability of the protein to bind P-selectin [3, 4, 7, 22, 23], here we saw that PSGL-1 was capable of binding both P- and L-selectin on all of our model viruses, regardless of the cell type used for virus production. However, robust E-selectin capture was only present on viruses from primary PBMC. These findings provide the basis for further studies to explore this role of PSGL-1 on virions within diverse tissue reservoirs, in ex vivo models and in vivo studies. Importantly, several other host proteins that have been shown to be virion-incorporated, such as ICAM-1 and integrin α4β7, are also known to maintain their functional activity and to be able to bind their cognate ligands and receptors, respectively [17, 24]. Likewise, both of these proteins have been shown to alter HIV-1 infection, either in mouse models [24] or ex vivo human tissues [46, 58].

Since P-selectin is known to be expressed in vivo on endothelial tissues [5, 59, 60] and is important in recruiting HIV-1 target cells like CD4 + T cells into intestinal tissues [61], it is reasonable to speculate that PSGL-1 present on virions could also mediate binding and extravasation of virions into inflamed tissues, similar to what occurs with PSGL-1-expressing leukocytes [4, 62]. Additionally, it is tempting to consider the possibility that HIV-1 virions may exploit incorporated-PSGL-1 to target intestinal CD4 + T cell populations and fuel local viral spread, similar to what was shown with integrin α4β7 in our previous work [24]. This proposed function of PSGL-1 as a viral homing molecule would be especially relevant for HIV-1 pathogenicity during the early phases of infection, when the gut is still populated with a large reservoir of target CD4 + T cells [63]. However, this hypothesis remains untested and more targeted experiments which more definitively assess the ability of PSGL-1 on virions to facilitate trans-infection should be the scope of future studies. Regardless of these outcomes, it is important to consider that functional PSGL-1 on virions may have uncharacterized impacts in vivo, contrary to the predominant inhibitory properties that PSGL-1 was previously shown to exert in vitro.

Lastly, while this work contributes to a better understanding of the role of PSGL-1 in HIV-1 infection, this protein may also have uncharacterized impacts on other microbial pathogens. Our work opens the field for new paradigms on how PSGL-1 can impact microbial infections, and expands the recently established paradigm that PSGL-1 is solely a host-restriction factor against HIV-1 viruses. The recent identification of PSGL-1 as a virion-incorporated restriction factor has fuelled a rapidly expanding and exciting field of literature. In this work we show that PSGL-1 on virions retains its ability to bind P-selectin and that through this process, virus with endogenous levels of PSGL-1 can be transferred to bystander cells for infection. These findings suggests that PSGL-1 could have additional roles in HIV infection beyond its previously described antiviral function.

As a rapidly evolving pathogen, it is unsurprising that HIV would be able to exploit a host restriction factor to enhance its pathogenesis. Our work underscores the importance of studying virion-incorporated proteins using a more holistic approach, as the impact of altered virus binding and virus restriction afforded by virion-incorporated PSGL-1 are likely not mutually exclusive. Future studies are warranted to establish the in vivo role of virion-incorporated PSGL-1 in virus homing and trans-infection, which could pave the way toward the development of novel antiviral therapies.

### Conclusions

While the antiviral effects of PSGL-1 in viruses produced by transfection is profound, HIV-1 continues to remain infectious when produced through natural infection of T cells and PBMC, even when PSGL-1 is incorporated. We found that pseudoviruses engineered with moderate amounts of PSGL-1 pDNA via transfection (25–250 ng) contained very high levels of incorporated PSGL-1, and they were much less infectious than viruses propagated in PBMC. Thus, our work confirms prior studies characterizing the potent antiviral effect of virion-incorporated PSGL-1 in HIV infection. We also found that PSGL-1 was consistently detected on a broad range of HIV-1 isolates, including viruses found in plasma from HIV-infected patients. Notably, we show that endogenous levels of virion-incorporated PSGL-1 can allow for PSGL-1-mediated virus capture and transfer to HIV-permissive host cells, via virus binding to P-selectin. Unique to this study are our findings which show PSGL-1-based virus capture and transfer, the first set of data to suggest that PSGL-1 may also work to mediate HIV-1 infection.

## Materials and methods

### Cell culture

The HEK293T and TZM-bl cell lines used to produce viruses and readout HIV infection, respectively, were obtained from the NIH HIV Reagent Program (ARP; Cat#103 and 8129) and were maintained in complete media comprised of DMEM (Wisent, Cat#319-005-CL), 10% fetal bovine serum (FBS; Wisent, Cat#098150), 100 U/mL penicillin, and 100 µg/mL streptomycin (Life Technologies, Cat#15140122). The H9, Jurkat (E6-1), PM1 and A3R5.7T cell lines (ARP, Cat#87, 177, 3038, 12386) and peripheral blood mononuclear cells used to produce virus through infection were maintained in RPMI-1640 (Wisent, Cat#350-000-CL) with the same components listed above. Recombinant human IL-2 (25 U/mL) was also added to PBMC cultures. All cells were grown in a 5% CO_2_ humidified incubator at 37 °C.

### Virus production via infection

Whole blood from healthy donors was collected in heparinized vacutainers (BD Biosciences) and PBMCs were subsquently isolated using density centrifugation with Lymphoprep (StemCell Technologies, Cat#07861). PBMCs were activated with 1% phytohemagglutinin (Gibco, Cat#LS10576015) and 50 U/mL of IL-2 in complete RPMI media for 72 h prior to infection with primary HIV isolates. T cell lines (H9, Jurkat, PM1, A3R5.7) or activated PBMC were pelleted and resuspended in 1 mL of replication-competent HIV isolates for 4 h to facilitate infection before fresh media was added to the cells. Cell culture supernatants containing virus were harvested 6–12 days later based on viral titre.

### Virus production via transfection

HIV pseudoviruses and replication-competent viruses (IMC) were produced with 2 μg of SG3^ΔEnv^ or pNL4-3 pDNA respectively. HIV-1 BaL.01 Env was used for pseudovirus to permit single round infection. For both pseudoviruses and replication-competent viruses, various levels of PSGL-1 pDNA were used (as outlined in Additional file 1: Table S1 and Fig. S4) to generate PSGL-1 negative, low, medium or high viruses (PSGL-1^Neg^, PSGL-1^Low^, PSGL-1^Med^, PSGL-1^High^). Where indicated, viruses that were produced with lower levels of PSGL-1 (0.025, 0.25 ng pDNA) had additional plasmid from an empty vector added to reach a total of 3 μg of pDNA. Both PV and IMC viruses were produced in HEK293T using Polyjet In Vitro Transfection Reagent (FroggaBio, Cat#SL100688). HEK293T cells were seeded at a density of 10^6^ cells/mL in 6-well plates in complete media and were transfected with 3 μg of pDNA after cells had reached 70% confluence. All transfections were performed with a 1:3 ratio of plasmid DNA (μg) to transfection reagent (μL). Six hours after transfection, the medium was replaced with complete DMEM to discard any viral progeny without incorporated host proteins. Cell culture supernatants containing virus were harvested 48 h after transfection. All HIV-1 expression vectors were acquired from the NIH ARP. The negative control and PSGL-1 expression vectors were obtained from Sino Biological (Cat#CV011 and HG10490-UT).

### Virus titration

For infection, TZM-bl cells were seeded into 96-well flat-bottom plates at 15,000 cells/well in 100 μL of complete DMEM. Virus stocks were added to the cells, yielding a total volume of 200 μL/well. Viral input was normalized by p24 for all viruses. Luciferase expression was detected 2 days later after removal of 135 μL of medium and the addition of 45 μL per well of Britelite™ Plus reporter assay (PerkinElmer, Cat#6066766). The cell lysates were transferred to Perkin Elmer Optiplates after 10 min for luminescence endpoint readout on a Synergy neo2 (BioTek) microplate reader. All samples were tested in duplicate wells.

### Antibodies

The following mouse and human monoclonal antibodies were utilized for virion capture assays: anti-PSGL-1 and mouse IgG1 isotype control (BD Biosciences, Cat#556053 and 557273) and anti-gp120s (clones 2G12 and PG9 acquired from the NIH ARP; Cat#1476 and 12149). For virion capture assays in patient plasmas, we used biotinylated antibodies, including CD44-bio (Miltenyi, Cat#130-113-340) and an in-house biotin conjugation (Bio-Rad Laboratories, Cat#LNK262B) of anti-PSGL-1 and mouse IgG1 isotype control (R&D, Cat#MAB002) antibodies. PE-conjugated mouse anti-PSGL-1 mAb (BD Biosciences, Cat#556055) was used for flow virometry labelling.

### Flow virometry

Flow virometry was performed using a Beckman Coulter CytoFLEX S with standard optical configuration and volumetric calibrations were performed as described previously [28]. Gain and threshold optimization for detection of virus and calibration beads was performed as described previously [64], with a modification of PE gain to 3000. All virus samples and controls were acquired at a sample flow rate of 10 μL/min for 1–2 min. Serial dilutions of select stained viruses were acquired on the cytometer to determine the presence of viral aggregates and to control for coincidence (Additional file 2: Serial dilutions excel sheet). For direct labelling, cell-free supernatants containing virus were diluted to 10^9^ particles/mL, or used undiluted if they were estimated to be less concentrated based on viral p24 data and stained overnight at 4 °C with a PE-conjugated mAb against PSGL-1. For labelling, 0.25 or 0.4 μg/mL of antibody was used on the viruses produced from infection and transfection respectively. These concentrations were determined empirically to maximize signal from specific labelling while minimizing levels of background fluorescence. After labelling, all samples were further diluted with PBS (to reduce coincidence) for analysis by flow virometry. Virus particle concentrations were determined by flow cytometry by gating on the virus population using SSC, as performed previously [28]. After staining virus stocks were diluted two-fold with 4% PFA (2% final) for 30 min for fixation. BD Quantibrite PE beads (CA, USA; Cat#340495, lot 47973) were used for fluorescence calibration, while NIST-traceable size standards (Thermo Fisher Scientific) were used for light scattering calibration (see Additional file 2 for full list). Light scatter calibration was performed using FCM_PASS_ software (https://nano.ccr.cancer.gov/fcmpass) as previously described [64, 65]. Detailed information on the fluorescent and light scatter calibration and the MIFlowCyt-EV checklist [66] can be found in the FCM_PASS_ output report in Additional file 2 along with the FCM_PASS_ data plots**.** All data were analyzed using FlowJo software version 10.7.1. (CA, USA). The median for PE MESF was generated using the gating strategy described the text using FlowJo.

### Virion capture assay

Immunomagnetic bead-based virion capture was performed as previously described [24, 32], with 25 μL of protein G Dynabeads (Life Technologies; Cat#10004D), which were armed with 0.5–1 μg of anti-PSGL-1, anti-gp120 antibodies or 0.5 μg of recombinant Fc chimera proteins (P/E/L-Selectin and MAdCAM-1; R&D Systems) for 30 min at room temperature and then washed with 10% FBS–PBS to remove unbound capture molecules. Where indicated the virus input was normalized at the same concentration using p24 across all viruses tested. For all tests equal virus volumes were used. Viruses were incubated with antibody-armed beads for 1–2 h at room temperature to allow virus capture. Beads were then washed twice with 10% FBS–PBS and once with 0.02% FBS–PBS to extensively remove unbound virus particles. The bead-associated virus was then treated with 0.5% Triton X-100 to lyse the captured virions for p24 quantification by AlphaLISA. Data analysis was performed using Prism v. 8.4.2 (GraphPad, San Diego, CA, USA). For capture using antibodies, the background level of virion capture for each virus type was assessed by virion capture with an isotype control antibody (as described above). The nominal level of background capture as detected using the non-specific isotype antibody was removed from each data point before graphing where indicated.

For experiments assessing viruses in plasma of HIV-infected patients, biotinylated antibodies (described above) were used to permit more sensitive virus capture assays with anti-biotin microbeads (Miltenyi, Cat#130-090-485). Levels of captured virus were measured by HIV-1 RNA detection via quantitative real-time PCR assays. Viral RNA was purified using the QIAamp Viral RNA kit (Qiagen) and the number of HIV-1 genome equivalents was obtained using previously reported primers, probe, and amplification conditions [67]. Plasma samples were contributed by Drs. Tae-Wook Chun and Susan Moir (patient ID #’s 3-5 and 8-12), and Dr. Frank Maldarelli (ID #’s 1-2 and 6-7).

### p24 AlphaLISA

The quantification of HIV-1 p24 capsid protein was performed in captured virus lysates and virus-containing supernatants with the high-sensitivity AlphaLISA p24 detection kit following the manufacturer’s (PerkinElmer) instructions. Absorbance readings were performed on a Synergy NEO 2 multimode plate reader (BioTek, VT, USA) equipped with Gen 5 software (v. 3.08).

### HeLa cell plate capture

HeLa cells were seeded in 24 well plates overnight to achieve a confluence of ~ 80%. The following day the cells were transfected with Polyjet transfection reagent following the manufacturer’s protocol with MAdCAM-1 or P-selectin plasmids. Protein expression was confirmed by cell staining and flow cytometry 24–48 h later. Following this the cells were washed 3X with warm DMEM before being blocked with 5% FBS in PBS at 37 °C for 30 min. After blocking, the wells were washed as before, and 300 μL of virus stocks were added to the wells for 2 h at RT. Virus stocks produced through transfection were normalized to allow for comparison between their differential PSGL-1 levels. Viruses produced in T cell lines were added to the wells at their undiluted titre. After viral incubation, the wells were washed to remove unbound virus as before and were treated with 0.5% triton X-100 in PBS to lyse captured virus. The viral lysates were assayed for p24 using AlphaLISA.

### Plate capture-transfer assay

Sterile tissue culture plates were coated overnight at 4 °C with 5 μg/mL recombinant P-selectin (R&D Systems, #137-PS-050) or MAdCAM-1 (R&D Systems, #6056-MC-050) proteins. The following day the wells were washed 3X with PBS before being blocked with 1% BSA in PBS at room temperature (RT) for 1 h. Following blocking, the wells were washed as before, and virus stocks were added to the wells for 2 h at RT. For IMC viruses generated through transfection the viral input was normalized by p24 while PBMC and T cell line viruses were used at their undiluted titre. After viral incubation, the wells were washed to remove unbound virus and were either treated with 0.5% triton X-100 in PBS to lyse captured virus or 15,000 TZM-bl cells were added to the wells to detect the ability of captured virus to be transferred to permissive cells. The viral lysates were assayed for p24 and TZM-bl cell luminescence was read 1–4 days later depending on the starting concentration of virus used. In brief, T cell line viruses that were of higher titre were incubated for 1–2 days, whereas viruses produced through transfection or infection of PBMC were incubated for 2–4 days to allow more time for replication.

### SDS-PAGE and immunoblotting

Virus-containing supernatants were concentrated using the PEG-it solution per the manufacturer’s (System Biosciences, Cat#LV810A-1) instructions. Concentrated viruses were lysed in RIPA buffer (BioBasic, Cat#RB4478) and denatured in Laemmli sample buffer (BioRad, Cat#1610747) with boiling at 95 °C for 10 min. 20 ng of p24/lane was separated on a 1 mm SDS-PAGE gel (4% stacking, 8% resolving) using the BioRad Mini-Protean system with 1X running buffer (BioRad, Cat#1610772). Proteins were transferred onto PVDF membranes with 1X transfer buffer (BioRad, Cat#1610771) supplemented with 20% methanol. Membranes were blocked (5% skim milk in TBST) for 1 h at room temperature. Primary antibodies were diluted in TBST: anti-p24 clone AG3.0 (1:1500; ARP, Cat#4121) and mouse anti-human PSGL-1 (0.2 µg/mL; same clone as above). Following a 2.5 h incubation at room temperature, primary antibodies were removed and membranes were washed in TBST. HRP-conjugated secondary antibodies were diluted in TBST and incubated for 1 h at room temperature: goat anti-human (1:10 000; Sigma, Cat#AP112P), goat anti-mouse (1:10 000; Enzo Life Science, Cat#BML-SA204-0100). Membranes were washed and developed using enhanced chemiluminescence (FroggaBio, Cat#CCH365-B100). Imaging was done on Bio Rad’s ChemiDoc XRS + using the Image Lab software.

### Statistical analyses

Statistical analyses were performed with Prism 9.0 (GraphPad Software). Two-way ANOVA was used to test the significance between antibody capture in patient samples from different stages of infection. Unpaired, two-tailed t tests or Mann–Whitney tests were used to compare differences between two groups following normality testing where indicated. Applied statistical analyses are indicated in the figure legends. Data are presented as mean ± SEM of three replicates unless stated otherwise in the figure legend.

### Flow cytometry

Flow cytometry used to assess cell surface expression of PSGL-1 was performed using a BD LSRFortessa (San Jose, CA, USA) instrument with FACS Diva software (San Jose, CA, USA), and all data were analyzed using FlowJo software version 10.7.1. (San Jose, CA, USA). T cell lines or activated PBMC were stained before infection with an isotype control antibody or a mouse anti-human monoclonal antibody against PSGL-1 (same clone as above) for 45 min. After primary antibodies were removed by washing, staining with an R-phycoerythrin (PE) conjugated F(ab’) 2-goat anti-mouse IgG secondary antibody (Invitrogen, Carlsbad, CA, USA; Cat#A10543) was performed for 20 min. HEK293T cells were stained following the same protocol 48 h after transfection. All staining was performed with 2 µg/mL of antibody in 4 °C in the dark.

## Supplementary Information

**Additional file 1: Table S1. **Amounts of plasmid DNA used to produce the BaL pseudovirus (PV) stocks. **Table S2.** Additional details regarding patient characteristics and the percentage of virion capture from Figure 5. **Figure S1.** PSGL-1 flow virometry staining quantification. Median PE MESF values from anti-PSGL-1 staining of viruses from Fig. 4. The MESF values from stained media controls, which demonstrate background levels of antibody fluorescence based on the detection limit for this instrument, were subtracted from all virus samples. Data shown are the mean + SD of three technical replicates. **Figure S2.** Determining the minimum threshold of detection for PSGL-1 staining on HIV-1 pseudoviruses using flow virometry. Staining of pseudoviruses produced through transfection with different amounts of PSGL-1 plasmid DNA (0–2.5 ng) with a PE-conjugated anti-PSGL-1 antibody. The horizontal dotted line on the virus dot plots denotes background fluorescence and the limit of instrument detection (~ 10 MESF). **Figure S3.** Detecting PSGL-1-incorporation on the surface of viruses and extracellular vesicles using flow virometry. **A** Staining of PSGL-1 positive viruses or matched cell culture supernatants (EV) from HEK293T cells transfected with different amounts of PSGL-1 pDNA (0 ng, 2.5 ng, 25 ng, 250 ng for negative, low, medium, high phenotypes respectively) with a PE-conjugated anti-PSGL-1 antibody. Virus and EV conditions are shown together to allow for comparison of the differential staining profiles. All conditions were transfected with an empty vector control to ensure the final amount of pDNA reached 3 μg. **B** PSGL-1 staining of viruses or vesicles from cell culture supernatants of infected (Virus) or mock infected (EV) T cell lines (H9, Jurkat, A3R5.7 and PM1). **C** PSGL-1 staining of infected or mock infected PBMC cell culture supernatants in two independent donors (D1–D2). The horizontal dotted line on the dot plots denotes background fluorescence and the limit of instrument detection (~ 10 MESF). Thick gates demarcate where the virus population is expected to be present based on light scattering. Events within the thin gate in the upper right quadrant were used to generate the histograms in each panel which display the comparison of the median light scattering properties of the EV and virus populations. Coloured histograms demonstrate the notable differences between virus (red) and EV (blue) populations and light scattering properties. **Figure S4.** Quality control assessment of the replication competent NL4-3 HIV produced through transfection with an infectious molecular clone. **A** Plasmid DNA concentrations used to produce viruses with distinct PSGL-1 phenotypes through transfection of HEK293T cells. **B** Both NL4-3 viruses (negative and high) were normalized based on viral p24 (displayed as 1:1 in graph) and were subjected to three-fold serial dilutions before incubation with the TZM-bl reporter cells for 48 h to measure viral infectivity. **C** Semi-quantitative comparisons of virion-incorporated PSGL-1 and gp120 on virus stocks using immunomagnetic virion capture assay as used in Fig. 3. **D** Staining of viruses for PSGL-1 incorporation using flow virometry with a PE-conjugated anti-PSGL-1 antibody. The horizontal dotted line on virus dot plots indicates background fluorescence and the limit of instrument detection (10 MESF). Results are representative of three independent experiments. For quantitative data, results shown are the mean ± SEM. **Figure S5.** PSGL-1+ virions can be captured by an anti-PSGL-1 mAb and transferred to HIV-permissive cells for infection. **A** Schematic depicting the experimental workflow: Viruses were added to wells pre-coated with monoclonal antibodies specific for either PSGL-1 or gp120, or with an isotype control (IgG) for two hours at room temperature to allow for virus binding. Post-incubation, wells were washed extensively to remove unbound virus and then were either assayed for the amount of captured virus using p24 detection (top workflow), or TZM-bl cells were overlayed onto each well containing captured virus, and trans-infection was measured via luminescence readout (bottom workflow). **B** Experimental results showing the levels of plate-based antibody-mediated virus capture and **C** viral infectivity from trans-infection assays. Only HIV IIIB viruses propagated in T cell lines (H9 in green or Jurkat in purple) were used for these proof-of-principle assays. Results are representative of three independent experiments and are displayed as mean ± SEM from three experiments with samples tested in duplicate.

**Additional file 2: **FCMPass calibration data excel sheet.

## Data Availability

The flow cytometry files for this study are available at the online flow repository (flowrepository.org; Ref:FR-FCM-Z596). All other files are available from the corresponding author on reasonable request.

## Abbreviations

CD62P: P-selectin
ECD: Extracellular domain
Env/gp120: HIV envelope glycoprotein
EV: Extracellular vesicle
IMC: Infectious molecular clone
mAb: Monoclonal antibody
MAdCAM-1: Mucosal vascular addressin cell adhesion molecule 1
MESF: Molecules of equivalent soluble fluorophore
PBMC: Peripheral blood mononuclear cells
pDNA: Plasmid DNA
PE: R-phycoerythrin
PSGL-1/CD162: P-selectin glycoprotein ligand-1
PV: Pseudoviruses
