# Supplementary material for: P-selectin glycoprotein ligand-1 (PSGL-1/CD162) is incorporated into clinical HIV-1 isolates and can mediate virus capture and subsequent transfer to permissive cells
Source: Retrovirology. 2022 May 21;19:9. doi: 10.1186/s12977-022-00593-5 (PMC9123692; doi:10.1186/s12977-022-00593-5)
Supplement: Supplementary file 1 — Additional file 1: Table S1. Amounts of plasmid DNA used to produce the BaL pseudovirus (PV) stocks. Table S2. Additional details regarding patient characteristics and the percentage of virion capture from Figure 5. Figure S1. PSGL-1 flow virometry staining quantification. Median PE MESF values from anti-PSGL-1 staining of viruses from Fig. 4. The MESF values from stained media controls, which demonstrate background levels of antibody fluorescence based on the detection limit for this instrument, were subtracted from all virus samples. Data shown are the mean + SD of three technical replicates. Figure S2. Determining the minimum threshold of detection for PSGL-1 staining on HIV-1 pseudoviruses using flow virometry. Staining of pseudoviruses produced through transfection with different amounts of PSGL-1 plasmid DNA (0–2.5 ng) with a PE-conjugated anti-PSGL-1 antibody. The horizontal dotted line on the virus dot plots denotes background fluorescence and the limit of instrument detection (~ 10 MESF). Figure S3. Detecting PSGL-1-incorporation on the surface of viruses and extracellular vesicles using flow virometry. A Staining of PSGL-1 positive viruses or matched cell culture supernatants (EV) from HEK293T cells transfected with different amounts of PSGL-1 pDNA (0 ng, 2.5 ng, 25 ng, 250 ng for negative, low, medium, high phenotypes respectively) with a PE-conjugated anti-PSGL-1 antibody. Virus and EV conditions are shown together to allow for comparison of the differential staining profiles. All conditions were transfected with an empty vector control to ensure the final amount of pDNA reached 3 μg. B PSGL-1 staining of viruses or vesicles from cell culture supernatants of infected (Virus) or mock infected (EV) T cell lines (H9, Jurkat, A3R5.7 and PM1). C PSGL-1 staining of infected or mock infected PBMC cell culture supernatants in two independent donors (D1–D2). The horizontal dotted line on the dot plots denotes background fluorescence and the limit of instrument de [file 12977_2022_593_MOESM1_ESM.pdf]

Table S1. Amounts of plasmid DNA used to produce the BaL pseudovirus (PV) stocks.

		pDNA (µg)			
	Phenotype	SG3Δ^{Env}	PSGL-1	BaL.01 Env	Total
Pseudovirus (PV)	PSGL-1^{Neg}	2	0	1	3
	PSGL-1^{Low}	2	0.0025	0.9975	3
	PSGL-1^{Med}	2	0.025	0.975	3
	PSGL-1^{High}	2	0.25	0.75	3

Virus stocks with different amounts of virion-incorporated PSGL-1 (PSGL-1 negative, low, medium, or high) were generated through the transfection of different amounts of plasmid DNA (pDNA). All transfections included an even amount of total pDNA (3 µg), with minor fluctuations in Env pDNA to account for the differential amounts of PSGL-1 pDNA used.

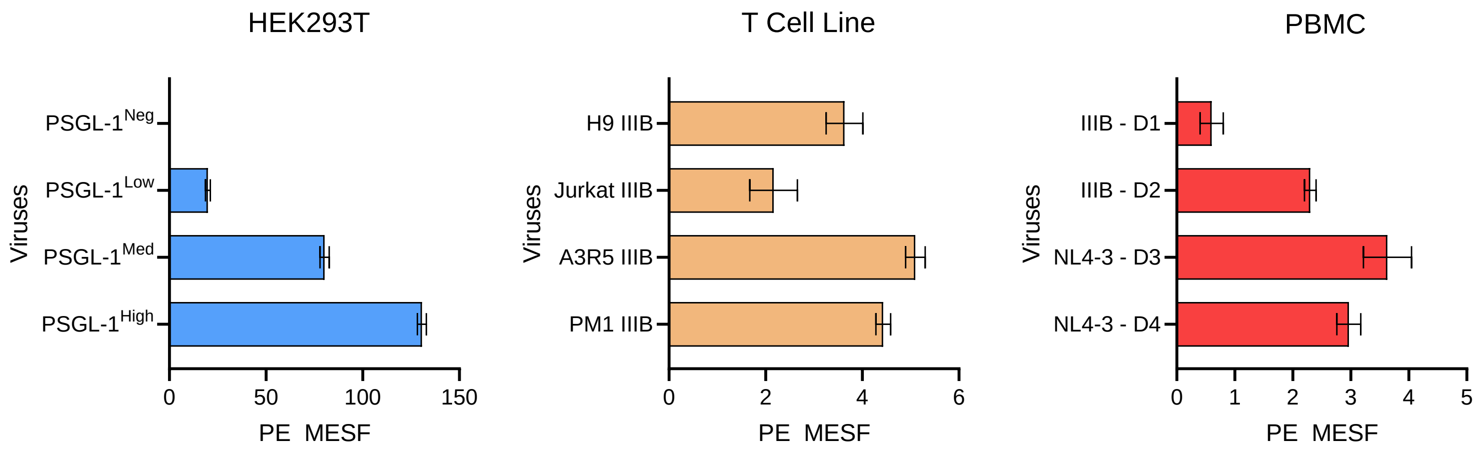

Figure S1. PSGL-1 flow virometry staining quantification. Median PE MESF values from anti-PSGL-1 staining of viruses from Fig. 4. The MESF values from stained media controls, which demonstrate background levels of antibody fluorescence based on the detection limit for this instrument, were subtracted from all virus samples. Data shown are the mean + SD of three technical replicates.

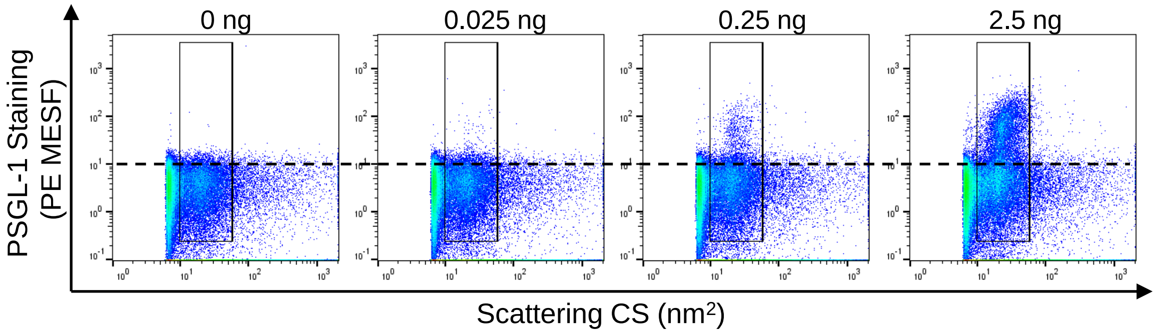

Figure S2. Determining the minimum threshold of detection for PSGL-1 staining on HIV-1 pseudoviruses using flow virometry. Staining of pseudoviruses produced through transfection with different amounts of PSGL-1 plasmid DNA (0-2.5 ng) with a PE-conjugated anti-PSGL-1 antibody. The horizontal dotted line on the virus dot plots denotes background fluorescence and the limit of instrument detection (~10 MESF).

Fig. S3A

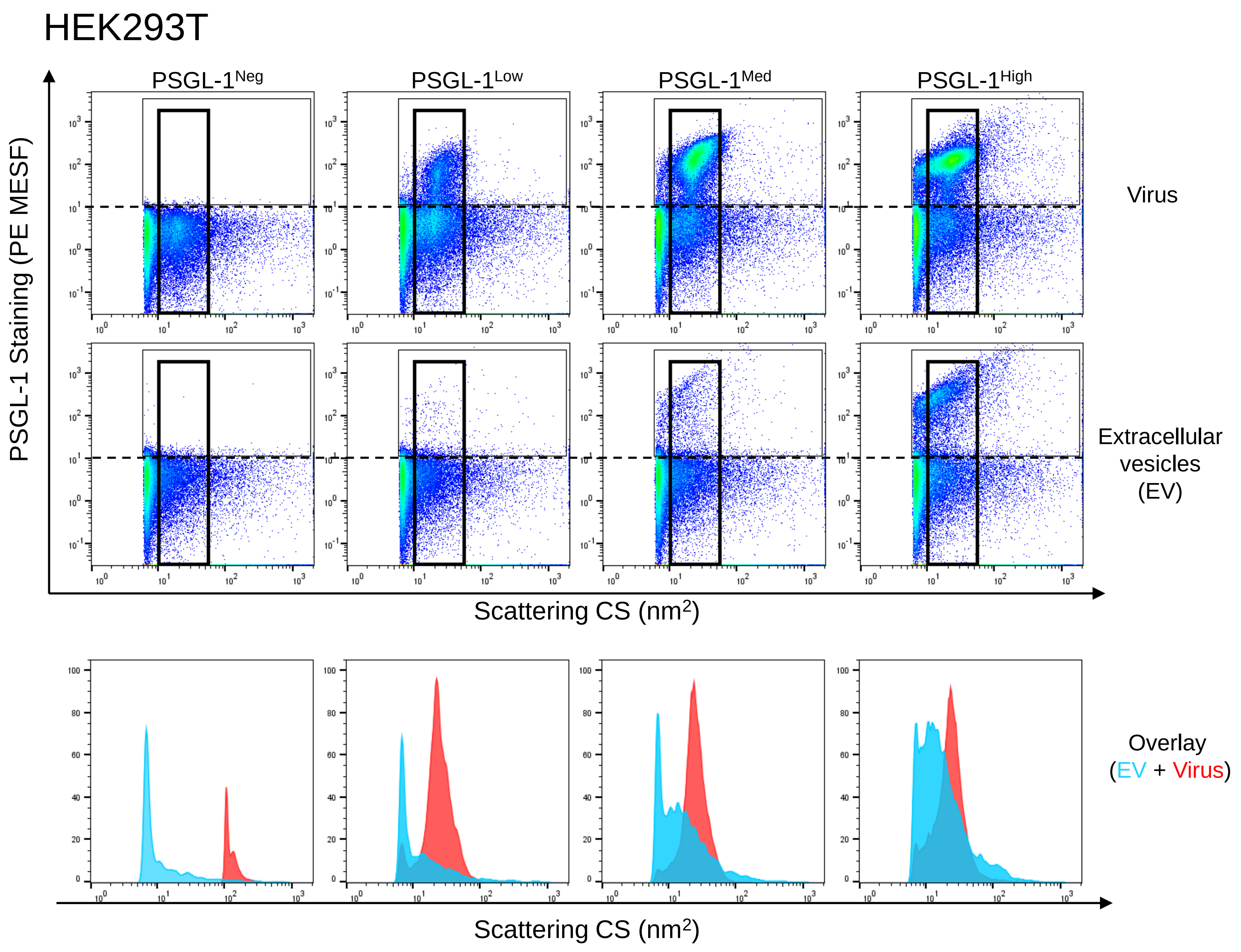

HEK293T
PSGL-1^Neg
PSGL-1^Low
PSGL-1^Med
PSGL-1^High
PSGL-1 Staining (PE MESF)
Virus
Extracellular vesicles (EV)
Scattering CS (nm²)
Overlay (EV + Virus)
Scattering CS (nm²)

Fig. S3B

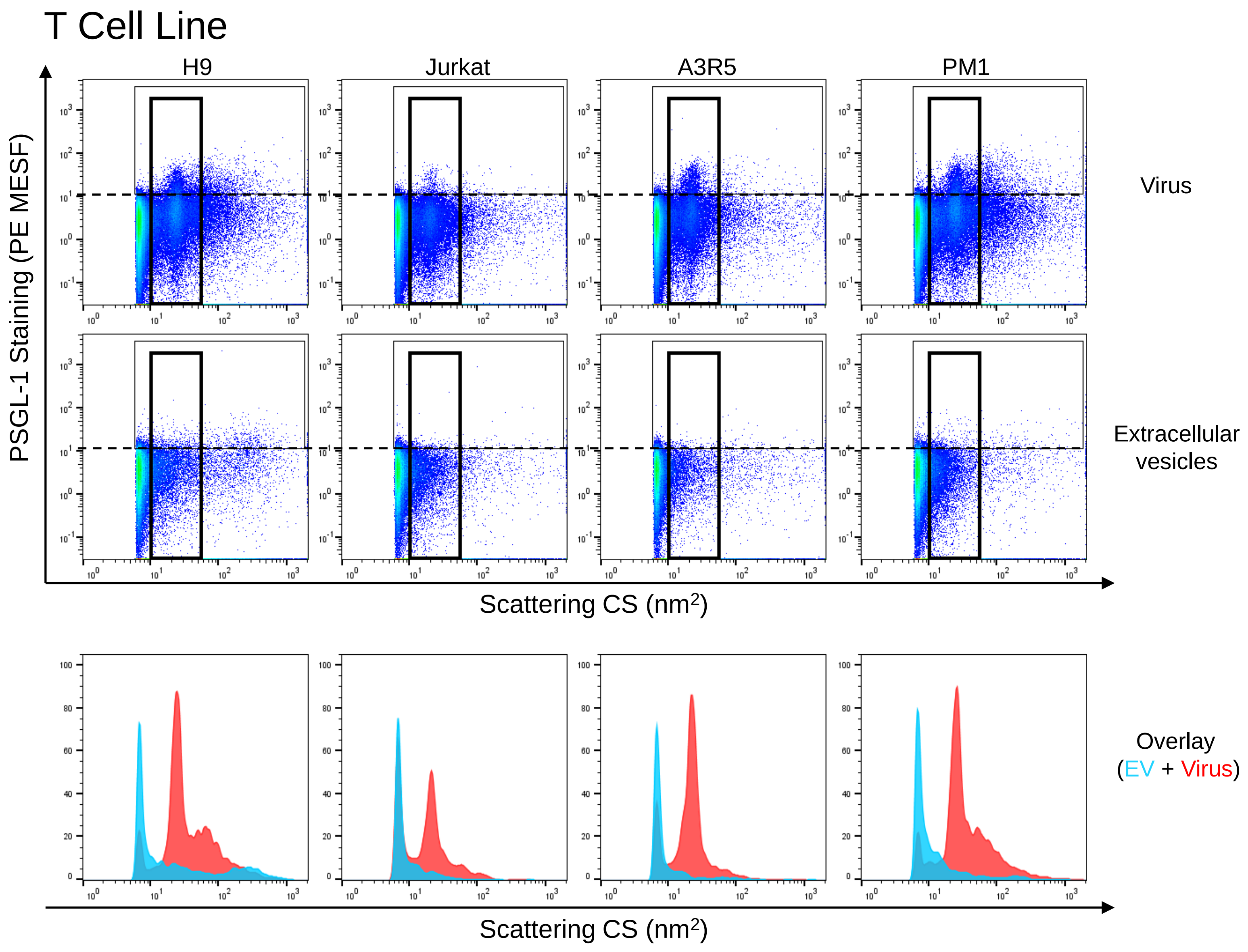
T Cell Line
H9
Jurkat
A3R5
PM1
PSGL-1 Staining (PE MESF)
Virus
Extracellular vesicles
Scattering CS (nm2)
Overlay
(EV + Virus)
Scattering CS (nm2)

Fig. S3C

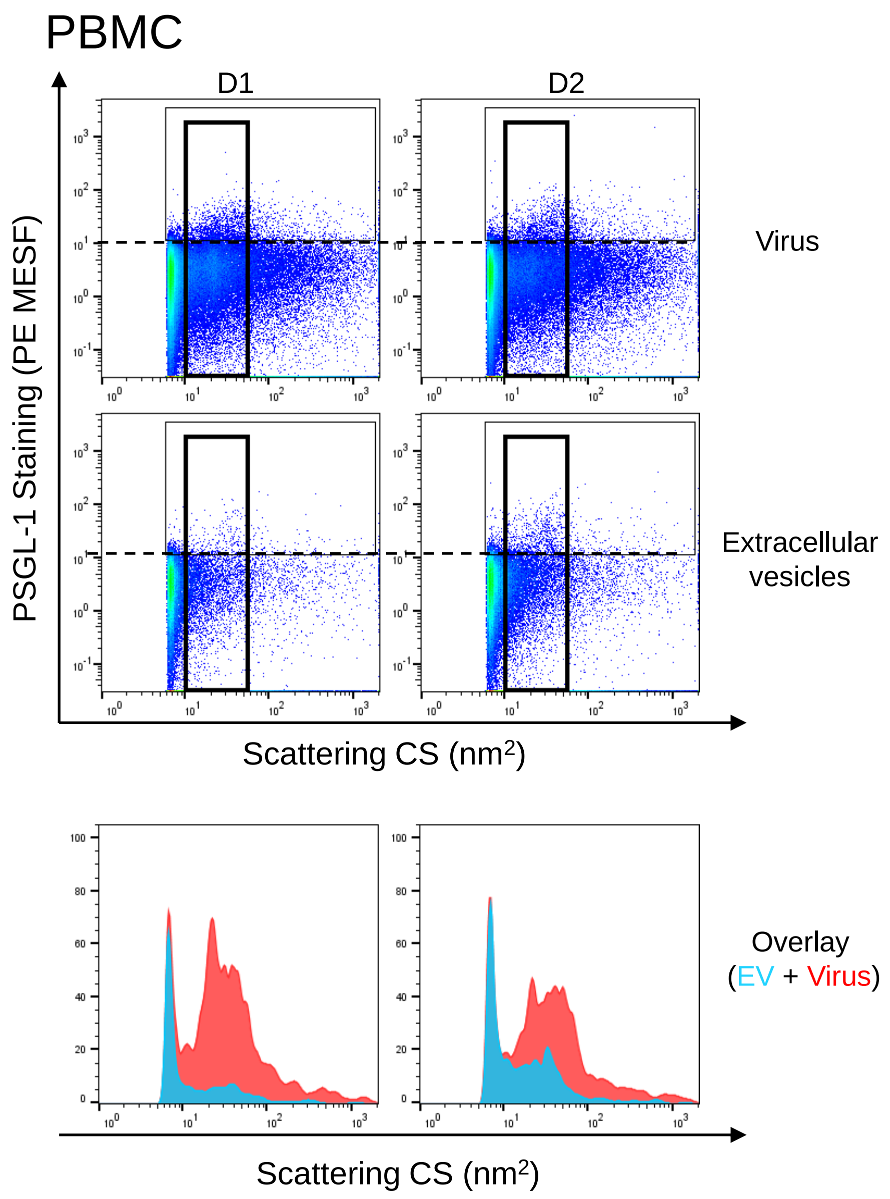

Figure S3. Detecting PSGL-1-incorporation on the surface of viruses and extracellular vesicles using flow virometry. (A) Staining of PSGL-1 positive viruses or matched cell culture supernatants (EV) from HEK293T cells transfected with different amounts of PSGL-1 pDNA (0 ng, 2.5 ng, 25ng, 250 ng for negative, low, medium, high phenotypes respectively) with a PE-conjugated anti-PSGL-1 antibody. Virus and EV conditions are shown together to allow for comparison of the differential staining profiles. All conditions were transfected with an empty vector control to ensure the final amount of pDNA reached 3 µg. (B) PSGL-1 staining of viruses or vesicles from cell culture supernatants of infected (Virus) or mock infected (EV) T cell lines (H9, Jurkat, A3R5.7 and PM1). (C) PSGL-1 staining of infected or mock infected PBMC cell culture supernatants in two independent donors (D1-D2). The horizontal dotted line on the dot plots denotes background fluorescence and the limit of instrument detection (~10 MESF). Thick gates demarcate where the virus population is expected to be present based on light scattering. Events within the thin gate in the upper right quadrant were used to generate the histograms in each panel which display the comparison of the median light scattering properties of the EV and virus populations. Coloured histograms demonstrate the notable differences between virus (red) and EV (blue) populations and light scattering properties.

Table S2. Additional details regarding patient characteristics and the percentage of virion capture from Figure 5.

			Captured Virus (% of Input)				
**Patient ID**	**Stage of Infection**	**Viral Load (copies/mL)**	**IgG Control**	**anti-PSGL-1**	**anti-CD44**	**CD4 count**	**CD8 count**
1	Acute/early	96530	5%	59%	46%	231	1822
2	Acute/early	93945	5%	75%	14%	670	1572
3	Acute/early	1755965	1%	30%	59%	842	573
4	Acute/early	17441	1%	44%	42%	448	1045
5	Acute/early	121659	2%	58%	60%	472	1667
6	Chronic	8701	47%	64%	64%	476	759
7	Chronic	82951	2%	44%	69%	580	957
8	Chronic	70426	6%	23%	36%	287	875
9	Chronic	102380	3%	33%	41%	527	1816
10	Chronic	60729	1%	34%	50%	445	1283
11	Chronic	48511	3%	50%	90%	559	1510
12	Chronic	106361	1%	60%	70%	254	424

A

		pDNA (µg)			
		NL4-3	PSGL-1	Empty vector	Total
Virus Phenotype	PSGL-1^{Neg}	2	0	1	3
	PSGL-1^{Low}	2	0.0025	0.9975	3
	PSGL-1^{High}	2	0.25	0.75	3

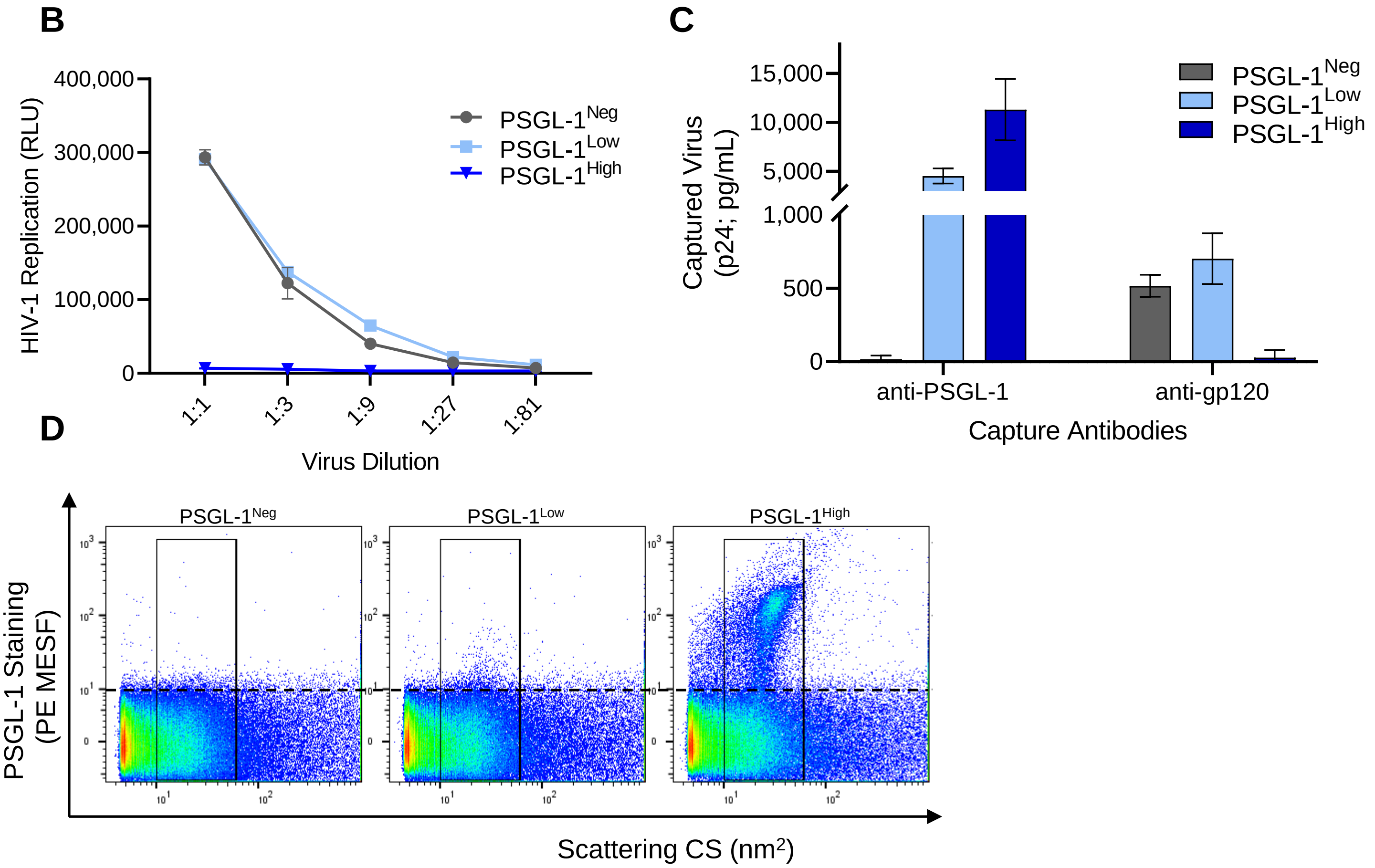

Figure S4. Quality control assessment of the replication competent NL4-3 HIV produced through transfection with an infectious molecular clone. (A) Plasmid DNA concentrations used to produce viruses with distinct PSGL-1 phenotypes through transfection of HEK293T cells. (B) Both NL4-3 viruses (negative and high) were normalized based on viral p24 (displayed as 1:1 in graph) and were subjected to 3-fold serial dilutions before incubation with the TZM-bl reporter cells for 48 hours to measure viral infectivity. (C) Semi-quantitative comparisons of virion-incorporated PSGL-1 and gp120 on virus stocks using immunomagnetic virion capture assay as used in Fig 3. (D) Staining of viruses for PSGL-1 incorporation using flow virometry with a PE-conjugated anti-PSGL-1 antibody. The horizontal dotted line on virus dot plots indicates background fluorescence and the limit of instrument detection (10 MESF). Results are representative of three independent experiments. For quantitative data, results shown are the mean ± SEM.

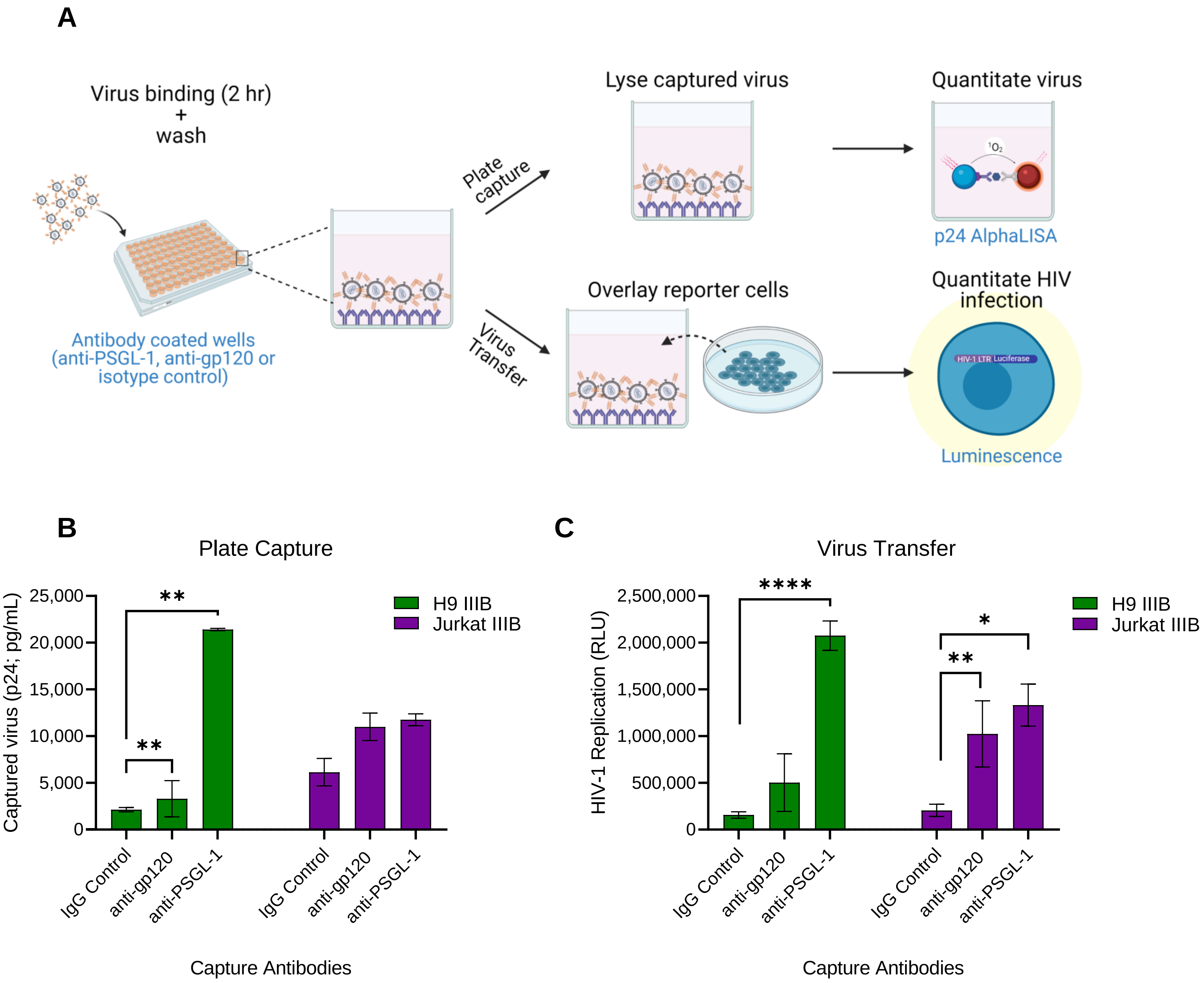

Figure S5. PSGL-1+ virions can be captured by an anti-PSGL-1 mAb and transferred to HIV-permissive cells for infection. (A) Schematic depicting the experimental workflow: Viruses were added to wells pre-coated with monoclonal antibodies specific for either PSGL-1 or gp120, or with an isotype control (IgG) for two hours at room temperature to allow for virus binding. Post-incubation, wells were washed extensively to remove unbound virus and then were either assayed for the amount of captured virus using p24 detection (top workflow), or TZM-bl cells were overlayed onto each well containing captured virus, and trans-infection was measured via luminescence readout (bottom workflow). (B) Experimental results showing the levels of plate-based antibody-mediated virus capture and (C) viral infectivity from trans-infection assays. Only HIV IIIB viruses propagated in T cell lines (H9 in green or Jurkat in purple) were used for these proof-of-principle assays. Results are representative of three independent experiments and are displayed as mean ± SEM from three experiments with samples tested in duplicate.
